# Patient support for tuberculosis patients in low-incidence countries: A systematic review

**DOI:** 10.1371/journal.pone.0205433

**Published:** 2018-10-10

**Authors:** Sarah van de Berg, Niesje Jansen-Aaldring, Gerard de Vries, Susan van den Hof

**Affiliations:** 1 KNCV Tuberculosis Foundation, The Hague, The Netherlands; 2 Center for Infectious Disease Control, National Institute for Public Health and the Environment (RIVM), Bilthoven, The Netherlands; 3 Dept. of Global Health, Academic Medical Center, and Amsterdam Institute for Global Health and Development, Amsterdam, The Netherlands; Indian Institute of Technology Delhi, INDIA

## Abstract

**Background:**

Patient support during tuberculosis treatment is expected to be more often available and more customized in low tuberculosis incidence, high-resource settings than in lower-resource settings. The aim of this systematic review is to provide an overview of tuberculosis patient support interventions implemented in low-incidence countries and an evaluation of their effects on treatment-related outcomes as well as their acceptability by patients and providers.

**Methods:**

PubMed, Social Science Citation Index and Cumulative Index to Nursing and Allied Health and Literature were searched for the period 01.2006–05.2016 on publications describing tuberculosis patient support interventions in low-incidence countries (<20 patients per 100,000 population).

**Results:**

Through our search strategy, 1875 unique publications were identified. Forty publications were included: 17 evaluated patient support quantitatively, 9 qualitatively and 14 only described the patient support. Nineteen publications assessed treatment supervision options only, 21 assessed (combinations of) treatment supervision, socio-economic, psycho-emotional, health-educational and other support. Of eight studies quantitatively evaluating the effects of support with a control group, four showed positive effects: two out of three that used combinations of patient support and two out of five that compared treatment supervision options. Heterogeneity of interventions precluded pooling of results. Qualitative and descriptive studies showed that patients appreciated individualized support including treatment supervision, psycho-emotional and socio-economic support; and digital health interventions.

**Conclusion:**

Our review shows that a variety of patient support interventions is implemented in low-incidence countries. Although only a few interventions were evaluated quantitatively, we identified potential best practices. The scarcity of evidence on effectiveness, however, indicates the need for further research to evaluate potential best practices.

## Introduction

Ensuring adherence to anti-tuberculosis (TB) treatment is a challenge, not only in high TB burden countries but also in countries with a low TB incidence and well-financed health care systems. Addressing barriers to TB diagnosis and treatment adherence for vulnerable and hard-to-reach groups are priority action areas for low-incidence countries progressing towards TB elimination. Detection of TB is high in most low-incidence countries through good access to care and enhanced case finding among risk groups [1]. Adherence to TB treatment, however, remains a challenge given the long treatment duration with multiple antibiotics; at least six months for drug-susceptible TB and 18–24 months for multidrug-resistant TB (MDR-TB), i.e. TB resistant to at least the two key first-line anti-TB drugs isoniazid and rifampicin [2,3]. Adverse drug reactions, early improvement of symptoms during treatment and socio-economic difficulties of the patient contribute to non-adherence to treatment, even in countries with relatively well-financed health care systems [4–11]. Interventions to ensure TB treatment adherence are not only essential in increasing the chance of cure but also in reducing the risk of further TB transmission and of drug resistance development [12–15].

There are various forms of patient support interventions to alleviate or remove barriers to treatment adherence including use of treatment supervision, also known as directly observed therapy (DOT), and other treatment administration support forms such as provision of pill boxes; health education; socio-economic support; and psycho-emotional support. Patient support interventions have been systematically reviewed and evaluated, but not yet specifically focussing on low-incidence countries [16–21]. Most of the studies reviewed, focused on high TB burden countries with limited financial resources or only on special risk groups. Patient support in low-incidence countries, however, may differ from support in high-incidence countries as in the former setting there usually are less patients and more resources. Patient support in high-resource countries may be more tailored to the individual patient’s needs and make more use of digital innovations, such as video observed treatment (VOT) [1,7]. In VOT medication intake is monitored through videos recorded by the patients or through video calls. In high-incidence countries interventions need to be provided to a larger number of patients representing a broader spectrum of the society, while resources are more limited.

In the Netherlands, all TB patients are entitled to support by a specialized TB nurse. This support may comprise health education, counselling, incentives and enablers. TB nurses also act as a case manager for the patient and coordinate the organization of treatment supervision, further socio-economic and psycho-emotional support. DOT can be provided by the TB nurse and/or a selected third party such as home nursing services. TB nurses determine the nature and intensity of support individually per patient based on an assessment at the start of treatment. For this assessment, the TB nurses interview patients in a structured way on disease-related factors, such as symptoms and co-morbidities, as well as on socio-economic and psycho-social factors. In the Netherlands, these factors are regarded essential for successful treatment. The patient should be well-informed (empowered), motivated to complete TB treatment, should be in financial and social stable living conditions, and side-effects and co-morbidities should be managed [22,23].

Treatment results in the Netherlands are satisfactory with 88% successful treatment outcome for rifampicin-sensitive TB for the years 2009–2013 [24]. As current patients support practices are not evidence-based, however, the question has been raised as to what are the most effective and efficient means of providing TB patient support. To develop an evidence-based Dutch guideline on patient support which may also be informative for other low-incidence countries, KNCV Tuberculosis Foundation initiated the project ‘Improving Patient Support Interventions’. This project includes a systematic review on evidence for patient support interventions in low-incidence countries, a European survey on countries’ patient support policies, and a qualitative study capturing the current Dutch practices.

### Objective

The aim of this review is to provide an overview of published TB patient support interventions in low-incidence countries, including their effectiveness in improving treatment adherence as well as their acceptability by patients and providers.

## Methods

### Eligibility criteria

As the aim of our systematic review was to identify evidence on patient support most relevant to low TB incidence settings such as the Netherlands, we only included publications describing TB patient support interventions in low-incidence countries [25]. Low incidence was defined as less than 20 per 100,000 population in line with the definitions of the European Centres of Disease Control [26]. Publications from all countries that had reached low TB incidence by 2014 were included [27]. Patient support interventions were defined as any intervention aiming to increase treatment adherence. Studies were excluded if they only compared adherence under different regimen options such as daily vs. thrice weekly doses. All publications measuring any qualitative or quantitative outcomes of patient support were included. Additionally, we included publications describing patient support as an ancillary intervention to the primary intervention under study, and the patient support component not being evaluated. Only articles published since 2006 were included as we aimed to capture the impact of recent evidence and practices, e.g. utilizing online interventions. We included publications in Dutch, English, German, Portuguese, Spanish, Italian and Russian.

#### Search strategy

The bibliographic database PubMed was searched on April 15^th,^ 2016 and the databases Cumulative Index to Nursing and Allied Health and Literature (CINAHL) and Social Science Citation Index (SSCI) were searched on May 2^nd,^ 2016 to identify relevant publications on patient support interventions. Search terms were developed by the authors and included combinations of three domains: (i) “tuberculosis” and related terms, (ii) “patient support interventions” and related terms and (iii) “treatment adherence” and related terms. A complete list of search terms is provided in S1 Appendix. Reference lists of relevant systematic reviews and of the included publications were scanned to identify additional publications.

Publications eligible for inclusion were selected in three steps: (i) screening of titles, (ii) assessment of abstracts and (iii) assessment of full texts. Titles and abstracts were assessed on eligibility independently by two researchers (SB and SH). Full text selection and review of reference lists was performed by one researcher (SB) and verified by a second reviewer (SH). Discrepancies between reviewers were resolved by discussion.

#### Data collection and analysis

Characteristics of patient support interventions were entered into a pre-piloted form. Extracted characteristics included the country where the intervention was provided, a description of the study design including, on availability, a description of the intervention and the control group. We divided patient support interventions into five categories: Treatment supervision, treatment administration support other than DOT, health educational (HE) support, socio-economic (SE) support and psycho-emotional (PE) support. These categories were defined as follows:

- **Treatment supervision:** Direct observation of medication intake by any person, at any location in any frequency, also known as directly observed therapy (DOT)
- **Treatment administration support other than DOT:** Tracer, pill boxes and other measures other than DOT to promote adherence
- **HE support:** Presentation of information to the patients aiming to educate them about their disease and its’ treatment as well as related health issues

- **SE support:** Provision of (social) services, material goods and/ or financial support
- **PE support:** Interventions focusing on the patients’ feelings, emotions or social relationships and social support

The following information was compiled on the interventions described in the publications: intervention categories included, reasons for patient support, patients eligible for the support, support provider(s), level of implementation (e.g. national/regional), duration of patient support provision, and experiences with provision. If available, also quantitative treatment adherence-related outcomes of the intervention were extracted. Treatment adherence-related outcomes as defined and calculated in the respective studies were adopted without changes.

#### Risk of bias in individual studies and quality of evidence

Risk of bias for studies quantitatively evaluating effects of patient support interventions against a comparison group was assessed using The Cochrane Collaboration’s Tool for Randomized Controlled Trials (RCT) for RCT and Newcastle Ottawa Scale (NOS) for Non-Randomized Studies (NRS). For NRS <10% of subjects lost was considered as indicative of low risk of attrition bias.

#### Summary measures and synthesis of results

Patient support interventions were described per category. Quantitative outcomes among patients receiving the interventions were described and, if applicable, compared to those among a comparison group of patients. Dichotomous outcomes were described using risk ratios (RR) for cohort studies and controlled trials and odds ratios (OR) for case-control studies, both with corresponding 95% confidence intervals. If not provided in the publication, ratios were calculated from the data provided in the publications, if possible. For non-dichotomous outcomes, absolute and relative differences (d_r_) were calculated. Ratios and relative differences were calculated using Microsoft Excel (2013).

## Results

### Publication selection

Through our search strategy, 2434 publications were identified of which 559 were duplicates and removed. From the remaining 1875 publications, 40 were eligible for inclusion (S1 Fig).

### Study characteristics

The 40 publications included 27 observational studies (cohort studies [28,29,38–41,30–37], case series [42–47], case-control studies [48–51], cross-sectional studies [9,52], and a costing study [53]); 9 descriptive qualitative studies (interview studies [54–59], a community-based ethnography [60], a focused ethnography [61], and an interpretive phenomenology [62]); 3 experimental studies (RCTs [63,64]), and a non-randomized controlled trial [65]) and 1 quasi-experimental study (historical before-after comparison) [66].

Publications were from the USA [29,31,53,64,65,35,36,41,42,44,46,47,50], the UK [9,43,51,54,57,61], Spain [30,32,38,40], Japan [52,56,59], Turkey [37,48,63], Canada [34,62], Australia [39], Greece [49], Italy [45], the Netherlands [28], New Zealand [60], Norway [58], Saudi Arabia [66] and Switzerland [33]. One study was multi-national, participating countries being Armenia, Australia, Central African Republic, India, Philippines, South Africa, Swaziland, Uganda and the UK [55]. Twenty-six studies evaluated patient support intervention qualitatively and/or quantitatively [30,35,49–51,54–60,38,61–66,39–41,43–45,48] (Table 1).

**Table 1:** Overview of studies quantitatively and/or qualitatively evaluating patient support in low tuberculosis incidence countries.

Study, Country	Study Type and Aim	Study Population	Support Categories Described				
			TS	HE support	SE support	PE support	Other^1^
**Studies quantitatively evaluating effects of patient support in comparison to a control group**^**2**^							
Babalık et al., 2013 [48], Turkey	Case-control study; Determine the factors influencing treatment outcomes and effectivity of the National Tuberculosis Program in relation to application of DOT	Adult TB patients with one year follow-up. Cases: adverse treatment outcome (n = 464), Controls: treatment outcome cured (n = 441); 92% on DOT	DOT at health care centres (50%), dispensaries (20%) and other (30%), provided by health care workers (76%) and other (24%)*	-	-	-	-
Caylà et al., 2009 [30], Spain	Prospective cohort study; Analyse anti-TB treatment adherence and fatality during standard TB treatments and identify factors associated with these event	Adult DS TB patients on standard anti-TB treatment. Exposed: patients on DOT^3^ (n = 140), Not exposed: patients on SAT (n = 1284)	DOT^#^^¥^	-	-	-	-
Chaudhry et al., 2015 [66], Saudi Arabia	Historical before-and-after study; Assess the effectiveness of the revised retrieval system (RRS) on non-compliance	Active PTB cases treated under DOT. IG: patients treated in 2005–2010 under RRS (n = 835), CG: patient treated in 2002–2004 before RRS (n = 501)	Out-patient DOT*^#^^¥^	RRS: For all patients education at admission and discharge; additionally, education at each OPD visit for substance-abusing patients^#^	-	-	RRS: For all patients follow up after missed OPD appointments by national TB control nurse; additionally, reminders by national TB control nurses one day prior to their appointments for substance-abusing patients
Chuck et al., 2016 [65], USA	Non-randomized controlled trial; Determine completion rates of VOT in comparison with in-person DOT, feasibility, acceptability and resource and staffing needs	(DR) TB patients eligible for DOT. IG: patients on VOT (n = 49); CG: patients on in-person DOT (n = 267)	VOT: Live videos of the patients via webcam-equipped computers^#^	-	-	-	Missed VOT appointments followed up by phone calls and home visits^#^
Clark et al., 2007 [63], Turkey	Prospective randomized study; Assess the effect of a clinical pharmacist directed patient education program (EDU) on the therapy adherence compared to routine nursing care	First-time TB patients on first-line anti-TB drugs. IG: EDU (n = 56); CG: no EDU (n = 58)	-	EDU: Oral and written education by clinical pharmacist shortly before discharge from the hospital	-	-	EDU: Appointment reminders by clinical pharmacist
King, Munsiff and Ahuja, 2010 [35], USA	Retrospective cohort study; Review treatment outcomes of HIV- positive TB patients in New York City and determinants for treatment success	HIV-positive, first-time, Rifampicin-sensitive TB patients. Exposed: patient on DOT^4^ (n = 1819), Not exposed: patients on SAT (n = 592)	DOT at home, worksite or another location convenient to the patient ^#^	-	-	-	-
Ricks et al., 2015 [64], USA	Randomized intervention study; Compare treatment outcomes using two different types of DOT outreach workers	Substance abusing active TB patient for which DOT was prescribed. IG: (n = 48), CG: (n = 46)	Enhanced DOT: DOT provided by peers in a two-person mixed-sex team^¥^	-	-	-	-
Wade et al., 2012 [39], Australia	Retrospective cohort study with CEA; Compare the effectiveness of in-person versus home videophone DOT (as measured by the proportion of appointments missed); to determine the cost-effectiveness of VOT; to determine acceptability, usability and sustainability of VOT	TB patients who had received VOT/DOT; Exposed: patient on VOT (n = 58), Not exposed: patients on DOT (n = 70)	VOT: DOT via desktop videophones and a call centre operating 24/7 and set up by a community nursing service	-	-	-	-
**Studies quantitatively evaluating effects of patient support without allowing for comparison of effects to a control group**^**2**^							
Charokopos et al., 2013 [49], Greece	Case-control study; Determine the effect of "modified DOT” (MDOT) on TB treatment outcomes, number of contacts tested for LTBI and number of contacts started on treatment in comparison to a SAT	Cases: newly diagnosed TB patients (n = 13) and close contacts (n = 30); Controls: past-treated TB patients (n = 41) and close contacts (n = 111)^5^	MDOT: Treatment supervision by GP during nine home visits, every 20 days	MDOT: Health education by the GP for the patient and household members during the visits	-	-	-
Craig et al., 2008 [43], UK	Case series; Develop a social outreach model of care including a TB link worker (TBLW) for marginalized groups with TB	Adult TB/LTBI patients referred on the basis of social need to TBLW (n = 100)	DOT at the DDU, at the pharmacy or the TB clinic*	-	TBLW: helps patients with challenging health and social care needs to access community services	-	-
Escudero et al., 2006 [40], Spain	Case series; Evaluate the results of the treatment of non-HIV-infected MDR-TB patients	HIV-negative MDR PTB patients (n = 25)	In-patient DOT by nurses	-	-	Psychological support and counselling by repeated clinical interviews on need and difficulties related to treatment adherence during hospitalisation and during out-patient follow-up	-
Ferrer et al., 2010 [41], USA	Case series; Report treatment outcomes among MDR-TB patients born in Mexico and treated along the US-Mexican border under a binational TB control project (Programa Juntos)	MDR-TB patients on DOT (n = 48)	Out-patient DOT by social workers^¥^	-	-	-	-
Garfein et al., 2015 [44], USA	Case series; Determine feasibility, acceptability, and potential efficacy of VOT in a high- and low-income setting	Adults newly diagnosed DS TB patients treated under VOT (n = 43 in San Diego; n = 9 in Tijuana)	VOT: Patients upload videos of themselves taking the medication to a cloud via a smart phone app^#^	-	-	-	Daily text message reminders (one before dose is due and one after the expected video had not been received)
Jit et al., 2011 [51], UK	Retrospective cohort study with CEA; Evaluate the cost-effectiveness of the Find and Treat Service for diagnosing and managing hard to reach individuals with active TB	Hard to reach individuals (e.g. homeless, substance abusing, imprisoned) a with active PTB. Cases: screened or managed by the Find and Treat service (n = 48), Controls: passively presenting controls (n = 252)^6^	-	Awareness raising events by Find and Treat Service supported by peer workers	-	Company to appointments by Find and Treat Service staff; home visits to reduce the risk of loss to follow-up	-
Luzzati et al., 2011 [45], Italy	Case series; Evaluate a prolonged hospitalisation programme to improve early outcome of TB treatment in high risk patients	Adult patients admitted to referral TB Centre for high risk (DR-TB, foreign born, illegal immigrant, previously treated, IDU, HIV infected or in a social and/or familiar condition not assuring good adherence to treatment) with positive smear culture-confirmed PTB (n = 122); 100% on DOT	In-patient DOT, subsequently out-patient DOT^#^^¥^	-	-	-	-
Mejuto et al., 2010 [38], Spain	Retrospective cohort study; Assess character, results and effectiveness of DOTS in the regional health area of Santiago de Compostela	TB patients who received DOTS treatment (n = 253)	DOT at TB unit, health centre, social services, family, DDU, school, hospital^#^	-	-	-	-
Pursnani et al., 2014 [50], USA	Case-control study nested in a retrospective cohort study; Compare patients undergoing court-ordered detention for TB treatment and time-matched control TB patients on outpatient DOT	Cases: Patients undergoing court-ordered detention for TB treatment (n = 79)^7^; Controls patients on outpatient DOT (n = 70)	Out-patient DOT*^#^^¥^	-	-	-	-
**Studies qualitatively assessing different aspects of patient support**							
Bender et al., 2011 [62], Canada	Interpretive phenomenology; Understand the nature of TB nurses’ relational work	Female nurses (n = 9) and their clients (n = 24)	DOT by nurses at patients’ homes, nurses’ cars, the street and other public settings*	Nurses repeatedly explain and clarify treatment plan	Incentives (such as grocery vouchers and public transit tokens)*	Nurses build rapport, encourage adherence without being authoritarian	-
Craig and Zumla, 2015 [54], UK	Interview study; Describe the social context of adherence to treatment in marginalized groups	Patients from a major TB centre (n = 17); 53% on DOT	DOT at the DDU, the pharmacy in conjunction with methadone and at hostels via outreach workers*	-	-	Outreach workers accompany patients to appointments*	Outreach workers provide appointment reminders*
Gerrish, Naisby and Ismail, 2013 [61], UK	Focused ethnography; Explore experiences of the diagnosis and management of TB from the perspective of Somali patients living in the UK and healthcare professionals involved in their care	Healthcare practitioners with experience of caring for Somali TB patients (n = 18), Somalis who had received TB treatment in the UK (n = 14)	-	-	Somali health care workers and TB nurses help patients to access other health and welfare services	-	-
Horter et al., 2014 [55], Multi-national	Interview study; Identify potential risks and benefits associated with blogging to determine whether social media had a role to play in supporting patients with MDR-TB	MDR-TB patient bloggers (n = 5); MSF project staff closely involved with the bloggers (n = 8); Stakeholders: WHO European Region TB specialists (n = 2) and members of staff from MSF headquarters (n = 5)	-	-	-	Blogging about MDR-TB treatment	-
Kawatsu et al., 2013 [56], Japan	Interview study; Explore the changes experienced by homeless TB patients and discuss the possible role of PHC-based DOTS treatment in effecting these changes	Ex-homeless TB patients who completed DOTS-based treatment at Shinjuku City PHC (n = 18)	DOT by nurses at the public health centre	-	Provision of food and drinks when patients come for DOT; nurses consult social welfare offices and other organizations	Nurses build rapport, address concerns, congratulation ceremony for successfully completed treatment	-
Mtui and Spence, 2014 [57], UK	Interview study; Explore the views and experiences of National Health Service (NHS) board TB nurses and consultants in public health medicine in relation to models of TB service delivery employed in their respective NHS boards in Scotland	TB specialist nurses (n = 6); health protection specialist nurse (n = 2); respiratory specialist nurse (n = 5); consultants in public health medicine (n = 5)	DOT at GP practices, in pharmacies for substance-abusers, on the streets / public bars by TB nurses for homeless patients	Nurses talk about TB and provide leaflets*	Nurses assist in accessing social care while delivering DOT; provide incentives for some cases, bring people to the clinic*	Nurses build rapport with patients, support in coping with the treatment, perform home visits*	-
Sagbakken, Bjune and Frich, 2011 [58], Norway	Interview study; Explore patients’ and health professionals’ views and experiences with DOT	Health professionals (n = 20), TB patients on DOT (n = 22)	DOT by homebased nursing services	-	-	-	-
Searle, Park and Littleton, 2007 [60], New Zealand	Community-based ethnography; Document and analyse the nature of the process of TB care in older European (Pakeha) TB patients	European TB patients in the Auckland region (n = 8); 63% on DOT	DOT at home by public health nurses*	-	Nurses ease structural constrains by arranging housing, food and transport	Nurses provide moral support and encouragements	-
Shimamura et al., 2010 [59], Japan	Interview study; Describe the support provided by Japanese public health nurses (PHN) to high-risk TB patients	PHNs (n = 11); patient cases described by the PHN (n = 11)	DOT by PHN*^¥^	PHN explain TB and co-morbidities to the patient and contacts, for patients with limited intelligence using a comic book or picture-story	PHN ensure physical place for homeless patients to receive medications, link patients with welfare service, build a support system for the future, including housing, food, or job training	PHN build rapport, encourage patients	Pill case provided for one patient with dementia who hoped to take her medicine independently

*target patients not specified

^#^provider not specified

^¥^DOT location not specified

^1^Treatment administration support other than DOT

^2^Including mixed-method studies

^3^Patients with high risk of low adherence (intravenous drug users, homeless, prisoners)

^4^Offered to all out-patients

^5^Publication does not provide treatment outcomes for controls precluding calculation of RRs

^6^Treatment outcome data based on modelling precluding calculation of RRs

^7^Patients undergoing court-ordered detention for TB treatment are not considered a comparison group for this systematic review as court-ordered detention is not considered patient support

CG: control group, CEA: cost effectiveness analysis, DDU: drug dependency unit, DOT: directly observed treatment, DR: drug resistant, DS: drug susceptible, GP: general practitioner, HE: health educational, HIV: Human Immunodeficiency Virus, IDU: injecting drug user, IG: intervention group, LTBI: latent TB infection, MDR-TB: multi-drug resistant TB, MSF: Médecins Sans Frontières, NHS: National Health Service, OPD: out-patient department, PE: psycho-emotional, PHC: public health centre, PHN: public health nurse, PTB: pulmonary TB, RRS: revised retrieval system, SAT: self-administered treatment, SE: socio-economic, TB: Tuberculosis, TBLW: TB link worker, TS: Treatment Supervision, VOT: video observed treatment

### Treatment support described

Of the 40 publications on TB patient support, 36 included treatment supervision options [9,28,39–48,30,49,50,52–54,56–60,31,62,64–67,32,33,35–38] (19 as the sole patient support intervention [9,29,39,41,42,45,48,50,58,64,30–33,35–38]), 12 included SE support [34,43,61,62,46,47,52,53,56,57,59,60] (1 as the sole intervention [61]), 11 included PE support [34,40,62,51,53–57,59,60] (1 as the sole intervention [55]), 8 included HE support [47,49,51,57,59,62,63,66] and 6 included treatment administration support other than DOT [44,54,59,63,65,66]. Nineteen publications described support packages of more than one patient support category [34,40,54,56,57,59,60,62,63,65,66,43,44,46,47,49,51–53]. An overview of the support described per category is provided in S2 Appendix.

Levels of intervention implementation, providers and target populations of patients support were specified in 33 [28,29,40,41,43–49,51,30,52–56,58,60–63,33,64–66,34–39], 20 [28,33,56–64,66,39–41,49,51–54] and 26 [28,30,46,47,49,51,52,54–58,34,59–61,63,64,66,35,38,40,41,43–45] publications, respectively (Table 1). Interventions were organized mostly on a local [29,34,62,63,35,36,38,43,47,48,51,61] or regional level [33,37,39,41,46,49,53,60,66]. Providers were mostly (n = 13) TB-, public health- or general nurses [28,39,61,62,66,40,52,53,56–60]. Most interventions (n = 20) were specifically aimed at patients at increased risk of non-adherence [28,30,52,54–57,59–61,64,66,34,38,40,41,43,45,46,51] such as substance abusing patients [30,38,66,45,46,51,52,54,57,59,64], homeless [30,34,51,52,56,57,59], MDR-TB patients [28,38,41,55], immigrants [38,45,61], and prisoners [30,51]. Six publications from Greece, Norway, the US and Turkey described interventions that were provided to all TB patients [35,44,47,49,58,63]. These interventions were mostly (n = 5) forms of DOT [35,44,47,49,58].

#### Treatment supervision options

DOT was described in 36 publications [9,28,37–46,29,47–50,52–54,56–58,30,59,60,62,64–66,31–36], and mostly (n = 27) comprised of out-patient DOT [29,33,46–50,52–54,56,57,34,58–60,62,64–66,35,36,38,39,41,43,44] provided at various locations, for example at the patient’s home [47,49,58,60], the pharmacy [33,43,52,54,57], the drug dependency unit [38,43,54], homeless shelters [46,54], on the streets or in public bars [46,57,62]. Out-patient DOT at the health centre was described in four publications [38,48,52,56]. Providers of DOT were mostly health care workers (n = 19) [1,9,53,54,56–60,62,66,28,33,34,39,40,48,49,52]. In one study, DOT was provided by social workers [41] and in another one by peers, who were former substance users [64].

VOT was described in four publications [36,39,44,65].

#### HE support and treatment administration support other than DOT

Treatment administration support other than DOT, mentioned in six publications, [44,54,59,63,65,66], was combined with HE support in four instances, and mostly (n = 5) included reminders by the treatment supporter and/or outreach workers [51,54,63,65,66]. One study described the provision of a pill case [59]. HE support, mentioned in eight publications [47,49,51,57,59,62,63,66], comprised in seven publications of health care workers involved in treatment supervision, mostly TB/ public health nurses, explaining about TB and its treatment [47,49,57,59,62,63,66]. One publication described awareness rising supported by peers [51].

#### SE- and PE Support

SE- and PE support were mentioned in 12 [34,43,61,62,46,47,52,53,56,57,59,60] and 11 [34,40,62,51,53–57,59,60] publications, respectively, of which 7 overlap [34,53,56,57,59,60,62]. The types of SE support described were material or directly provided enablers (n = 8) [34,46,47,56,57,59,60,62], help in accessing social or welfare services (n = 8) [43,47,53,56,57,59–61] and the provision of incentives (n = 6) [34,46,52,53,57,59] such as job trainings [59] and financial support [34,46,53]. In most publications (n = 9) SE support was part of the routine care health care workers provided to TB patients [34,47,53,56,57,59–62]. In one study, a dedicated “TB link worker” was responsible for helping patients in accessing social care [43]. PE support mostly (n = 5) comprised of TB/ public health nurse supervising treatment building rapport providing moral support for the patient [56,57,59,60,62]. Other PE support interventions included accompanying patients to (clinic) appointments [43,51,60], counselling by a clinician and a psychologist [40], a DOT completion ceremony [56] and blogging about treatment [55].

### Quantitative evidence on patient support described

Patient support is quantitatively evaluated in 17 studies; in 8 with comparison to a control group and in 9 without comparison to a control group.

#### Studies quantitatively evaluating patient support compared to a control group

Of the eight studies that compared a patient support intervention with a control group [30,35,39,48,63–66], five evaluated different forms of treatment supervision options [35,39,48,63–66], one evaluated treatment supervision combined with reminders[65]; and two evaluated provision of HE support combined with reminders [63,66]. In four studies, interventions were associated with a significant improvement in treatment adherence. Patients whose treatment was supervised by peers had a lower risk of treatment failure compared to patients whose treatment was supervised by health workers (RR = 1.40 [CI: 1.08–1.82]) [64]. Patients whose treatment was supervised at a location convenient to them were more likely to successfully complete treatment compared to patients on self-administered treatment (SAT) (RR = 1.14 [CI: 1.07–1.22]) [35]. Patients who received HE support combined with reminders were more likely to attend 100% of the follow-up visits and to complete treatment, respectively, compared to patients receiving routine care (RR = 1.83 [CI: 1.1–2.9]; RR = 1.16 [CI: 1.11–1.20]) [63,66]. In the other four studies [30,39,48,65], patient support interventions had no statistically significant effect on treatment adherence. In one of these studies, treatment outcomes were not influenced by the type of DOT provider, i.e. health workers versus other providers [48]. In two of these studies, VOT was not superior compared to in-person DOT in terms of treatment completion (RR = 1.49 [CI: 0.95–2.33]; RR = 0.99 [CI: 0.93–1.05]) [39,65]. VOT, however, did increase effectivity in terms of the number of successful observations (RR = 1.05 [CI: 1.04–1.06]; d_r_ (average number of non-observations) = -67%). DOT was also not superior compared to SAT in terms of less adverse treatment outcomes and less treatment default, respectively (RR = 1.12 [CI: 0.59–2.11]; OR = 1.37 [CI: 0.85–2.21]) [30,48]. An overview of the outcomes of the interventions is provided in Table 2.

**Table 2:** Quantitative outcomes and effects of tuberculosis patient support interventions in low-incidence countries described in studies allowing for comparison to a control group.

Source	Target group*		Intervention (comparison)	Outcome^1^	N	IG	CG		Effect (95% CI, p value)
Babalık et al., 2013 [48], Turkey	Adult TB patients with one year follow-up		DOT (SAT)	Adverse treatment outcome (default, death, and treatment failure)	905 (IG:830, CG:75)	431 (52%)	33 (44%)		OR^2^ = 1.37 [CI:0.85–2.21]
			DOT at the health care centre (DOT at the dispensary)		581 (IG:415, CG:166)	206 (50%)	93 (56%)		OR^3^ = 0.92 [CI:0.63–1.36]
			DOT at other locations (DOT at the dispensary)		415 (IG:249, CG:166)	132 (53%)	93 (56%)		OR^3^ = 0.88 [CI:0.57–1.36]
			SAT (DOT at the dispensary)		241 (IG:75, CG:166)	33 (44%)	93 (56%)		OR^3^ = 0.69 [CI:0.38–1.26]
			SAT (DOT by HCW)		702 (IG:75, CG:627)	33 (44%)	322 (51%)		OR^3^ = 0.73 [CI:0.43–1.23]
			DOT by others (DOT by HCW)		830 (IG:203, CG:627)	109 (54%)	322 (51%)		OR^3^ = 0.88 [CI:0.61–1.26]
Caylà et al., 2009 [30], Spain	TB Patients at high risk of low adherence		DOT (SAT)	Treatment default	1424 (IG:140, CG:1284)	10 (7%)	82 (6%)		OR^4^ = 1.12 [CI:0.57–2.22]
Chaudhry et al., 2015 [66], Saudi Arabia	Infectious PTB patients		Revised patient retrieval system (vs. baseline phase)	Treatment completion	1336 (IG:835, CG:501)	816 (98%)	423 (84%)		RR = 1.16 [CI:1.11–1.20], p<0.01
				Retrieval after missed appointments	239 (IG: 98, CG:141)	79 (81%)	63 (45%)		RR = 1.80 [CI:1.47–2.22], p<0.01
Chuck et al., 2016 [65], USA	Patients eligible for DOT		Synchronous VOT (vs. in-person clinic and community DOT)	Treatment completion	316 (IG:49, CG:267	47 (96%)	260 (97%)		RR = 0.99 [CI:0.93–1.05]
				Number of successful DOT sessions		3292 (95%)	32204 (91%)		RR = 1.05 [CI:1.04–1.06], p<0.01
Clark et al., 2007 [63], Turkey		First-time patients, newly diagnosed, receiving first-line drugs	Pharmacist-led patient education (vs. routine medical and nursing care)	Number of patients who attended 100% of the follow-up visits	114 (IG:56, CG:58)	30 (54%)		17 (29%)	RR = 1.83 [CI:1.1–2.9], p = 0.01
				Number of patients with 100% of isoniazid metabolites test results positive	103 (IG:51, CG:52)	41 (80%)		22 (42%)	RR = 1.90 [CI: 1.4–2.7], p<0.01
				Observed / expected doses taken		88.7%		85.8%	d_r_ = 3%, d_a_ = 2.9% [CI:-0.83–6.63] p = 0.13^#^
King, Munsiff and Ahuja, 2010 [35], USA		Patients treated with Rifabutin, on DOT voluntarily or due to non-adherence	Ever on DOT (never DOT)	Treatment success	2411 (IG:1819,CG:592)	1494 (82%)		325 (55%)	OR^5^ = 2.82 [CI:1.88–4.25]
Ricks et al., 2015 [64], USA		Substance abusing patients	DOT by Department of Public Health personnel (vs enhanced DOT)	Treatment failure	94 (IG:48, CC:46)	8 (15%)		18 (39%)	RR^6^ = 2.7 [CI:1.2–5.8] p = 0.01
				Mean number of treatment interruptions		1.4		4.5	d_r_ = -69% p = 0.06^¥^
				Mean treatment length of interruptions (measured by number of interruptions)		1.3		2.7	d_r_ = -52% p = 0. 42^¥^
Wade et al., 2012 [39], Australia		Patients eligible for DOT	VOT (vs. in person home and clinic DOT)	Treatment completion	115 (I:45, C:70)	22 (49%)		23 (33%)	RR = 1.49 [CI:0.95–2.33], p = 0.08
				Average number of non-observations		13.4		40.6	d_r_ = -67%, d_a_ = 27.5 [CI:16.6–40.0]
				Proportion of episodes not observed		12.1%		31.1%	d_r_ = 61%, d_a_ = 18.9% [CI:12.2–25.4]

*descriptions of the target groups can be found in S2 Appendix

^#^statistical analysis consisted of Student’s T-test

¥statistical analysis consisted of Wilcoxon rank-sum test

CG: control group, CI: Confidence Interval, d_a_: absolute difference, DOT: Directly Observed Treatment, d_r_: relative difference, IG: intervention group, N: number, OR: Odds Ratio, PTB: Pulmonary TB, RR: Risk Ratio, TB: Tuberculosis, VOT: Video observed treatment

^1^ Outcome as reported in the respective publication, may comprise desirable and undesirable outcomes

^2^ OR calculated based on data provided in the publication

^3^ Adjusted OR provided in the publication

^4^ OR provided in the publication

^5^ Adjusted OR provided in the publication

^6^ Adjusted RR provided in the publication

#### Studies quantitatively evaluating patient support without comparison to a control group

Of the nine studies that quantitatively evaluated patient support without comparison to a control group [40,41,43–45,49–51,68], seven evaluated different forms of DOT [40,41,44,45,49,50,68], one evaluated SE support [43] and one combined HE support with PE support and reminders [51] (Table 3). Under the different forms of DOT, treatment completion/ cure rates ranged from 70% to 89%. Treatment completion was 78% in one study in which SE support was provided to patients [43] and 61% in one study in which HE support combined with PE support and reminders was provided to patients [51].

**Table 3:** Quantitative outcomes of tuberculosis patient support interventions in low incidence countries described in studies not allowing for comparisons to a control group.

Source	Intervention	Target group*	N	Outcome	N outcome
Charokopos et al., 2013 [49], Greece	Modified DOT	Newly diagnosed TB patients	54	Treatment completion	11 (85%)
Craig et al., 2008 [43], UK	TB link worker	Adult TB/LTBI patients referred on the basis of social need to TBLW	90	Treatment completion	70 (78%)
Escudero et al., 2006 [40], Spain	In-patient DOT	HIV-negative MDR-TB patients	25	Treatment completion	21 (84%)
Ferrer et al., 2010 [41], USA	DOT by social worker	MDR-TB patients	46	Treatment completion	30 (65%)
Garfein et al., 2015 [44], USA	VOT via uploading videos via a smart phone app + daily text message reminders	Adult newly diagnosed DS TB patients treated under VOT	41	Treatment adherence [average doses missed]	2.7±7
				Treatment adherence [observed doses/ expected doses]	93%
Jit et al., 2011 [51], UK	Find and Treat Service	Hard to reach individuals with active PTB	188	Treatment completion^#^	61%
Luzzati et al., 2011 [45], Italy	In-patient DOT	Adult patients admitted to referral TB Centre for high risk	122	Treatment adherence [*not defined*]	96%
Mejuto et al., 2010 [38], Spain	DOT at various locations	TB patients who received DOTS treatment	253	Treatment completion	213 (82%)
Pursnani et al., 2014 [50], USA	Out-patient DOT	Patients on out-patient DOT	70	Treatment completion	62 (89%)

*descriptions of the target groups can be found in S2 Appendix

#Based on modelling data

DOT: Directly Observed Treatment, N: number, PTB: Pulmonary TB, TB: Tuberculosis, VOT: Video observed treatment

### Acceptability of patient support described

Thirteen studies qualitatively described how acceptable patient support was to patients and providers (Table 4). Patients appreciated individualized support by nurses [56,60,62] which included DOT at the patients home [60,62] or the health care centre [56] but also various forms of psycho-emotional support, such as nurses providing food and building rapport. Interventions appreciated by patients as well as providers included VOT [39,44,65], a “TB link worker” [43], blogging about TB [55], and Somali health care workers providing support, together with specialized TB nurses, for people of Somali origin living in the UK [61]. Patients appreciated VOT due to its convenience, privacy and flexibility [39,65]. Providers were satisfied with VOT due to travel time saved [44] and the ability to easily assess patients repeatedly [39]. Some obstacles were encountered by providers, which were mainly of a technical nature and patient-related challenges such as patients not following observation protocols [39,65]. The “TB link-worker” was generally appreciated by patients as they had to go only to one “stop” for both social and emotional support. After the introduction of the TB link worker, 88% of the patients achieved jointly set goals such as temporary housing and secured income. Stakeholders appreciated additional time and increased information and knowledge exchange with the clinical teams [43]. Blogging was perceived, both by patients and providers, as a tool to empower patients [55].

**Table 4:** Qualitative outcomes of and experiences with tuberculosis patient support interventions described in low incidence countries.

Study	Intervention	Outcomes
Bender et al., 2011 [62], Canada	TB nurses provide DOT at patients’ homes, nurses’ cars, the street and other public settings; Nurses repeatedly explain and clarify treatment plan; Nurses provide incentives; Nurses build rapport, encourage adherence without being authoritarian	Patients emphasized the emotional well-being that came from the way that nurses addressed fears, challenged the stigma of TB, and helped with other health concerns; In some cases, nurses felt like intruders; The dual surveillance-care focus of client visits required nurses to balance the intrusiveness of these visits with a welcoming and friendly approach providing comfort to the patient
Chuck et al., 2016 [65], USA	Synchronous VOT	Fifty-nine patients reported choosing VOT due to its convenience, four for privacy and one for flexibility; 346 VOT-related issues were identified for 54 patients (276 technical problems, 49 patient-related challenges such as patients forgetting their appointment, having schedule conflicts, or patient being out of camera view, 21 due to smartphone misuse)
Craig and Zumla, 2015 [54], UK	DOT is provided at the drug dependency unit, the pharmacy in conjunction with methadone and at hostels via outreach workers, Outreach workers accompany patients to appointments and provide appointment reminders	Patients felt resentment when DOT was provided in an authoritarian atmosphere; DOT was not always successful even when the location or provider was changed; Substance abusers did not always attend the drug dependency unit where DOT was provided due to travel distance or drug use; Quality of monitoring of pill swallowing varied across different healthcare locations; Outreach workers were not always reliable in providing reminders
Craig et al., 2008 [6], UK	TB link worker helps patients with challenging health and social care needs to access community services	The introduction of the TB link worker improved communication of out-patient and in-patient care providers, particularly in relation to hospital discharge, lead to additional time for care providers, increased information exchange and awareness of the disease among care provider, ensured patients received intensive emotional and practical support in a ‘one-stop-shop’ fashion which was an incentive for patients to engage with the services; Goals jointly agreed on by patient and TB link worker (concerning housing, immigration, income/benefits, treatment completion, DOT, drug- and alcohol support, criminal justice) were totally achieved for 57% (38/67) of patients and partially achieved for 31% (21/67), 3 patients refused assistance from the TB link worker; for 12 cases goals were not achieved because: patients did not contact the community services (n = 5), patients were not considered eligible to receive the service (n = 4), patients refused the housing offered to them (n = 2), there were no vacancies at the hostel (n = 1)
Garfein et al., 2015 [44], USA	Asynchronous VOT	Thirty-eight patients (92.7%) would choose VOT if repeat of anti-TB treatment was needed; All would recommend VOT to other TB patients; 24 (60%) found text message reminders helpful; Nurses reported that time and travel saved using VOT allowed them to concentrate on less adherent patients; VOT providers contacted patients to encourage adherence, provide re-training on VDOT procedures, and/or troubleshoot technical problems with recording videos. Older participants perceived experienced no barrier to using VDOT but enjoyed learning to use a smartphone and the autonomy related to VOT,
Gerrish, Naisby and Ismail, 2013 [61], UK	Somali health care workers and TB nurses help patients to access other health and welfare services	The support of TB specialist nurses and Somali health workers was highly valued by patients and healthcare professionals
Horter et al., 2014 [55], Multinational	Blogging about MDR-TB treatment	Patients mentioned blogging about MDR-TB treatment was supportive for adherence, considered blogging a tool to receive and provide peer support, a platform to express themselves and mean to record their achievement of which they can be proud; Stakeholders considered blogging a tool to provide treatment support to patients and to empower patients; Project staff and stakeholders considered blogging a tool to enhance patient practitioner relationships and to improve the understanding of the patient's experience with the disease; One blogger mentioned expectations of financial gain as a result of blogging
Kawatsu et al., 2013 [56], Japan	Nurses provide DOT at the public health centre, and food and drinks when patients come for DOT; Nurses consult social welfare offices and other organizations; Nurses build rapport, address concerns and organize congratulation ceremony for successfully completed treatment	Patient empowerment was achieved comprising the fulfilment of emotional needs, improved mental health, improved health behaviour, improvement of living environment, improved interpersonal relationships and improved attitudes towards society
Mtui and Spence, 2014 [57], UK	DOT is provided by the general practitioner; TB nurses provide DOT in pharmacies for substance-abusers, on the streets / public bars by TB nurses for homeless patients; Nurses talk about TB and provide leaflets; Nurses assist in accessing social care while delivering DOT, provide incentives for some cases and bring people to the clinic; Nurses build rapport with patients, support in coping with the treatment and perform home visits	Nurses reported that lengthy time for travel, long duration of visits, and high number in receipt of DOT were challenging; One National Health Service board reported threats to nurses from people known to patients while visiting; One National Health Service board reported problematic treatment adherence with the immigrant population due to fears of deportation
Sagbakken, Bjune and Frich, 2011 [58], Norway	DOT by homebased nursing services	Some patients had the experience of being cared for by DOT provision; Most patients experienced DOT as humiliating and discriminating as there was little room for patients to negotiate whether they consent to DOT, because DOT appointments could not be scheduled flexibly and because the health care worker proving DOT changed frequently
Searle, Park and Littleton, 2007 [60], New Zealand	DOT is provided at home by public health nurses; Nurses ease structural constrains by arranging housing, food and transport; Nurses provide moral support and encouragements	One patient appreciated the encouragement of the public health nurse and the monthly visits; One patient appreciated the nurse’s sensitivity regarding stigma; One patient appreciated the nurse’s flexibility in planning meetings around his business trips
Shimamura et al., 2010 [59], Japan	Pill case provided by public health nurse	For one patient with dementia who hoped to take her medicine independently a pill case created by the public health nurse facilitated adherence
Wade et al., 2012 [18], Australia	Synchronous VOT	Patients valued the convenience, flexibility and reliability and privacy of VOT, developed rapport with the nurses via VOT and found the technology was easy to use; Nurses reported that many more patients could be seen in a shift than with a drive-around service, more convenient scheduling was regarded as improving patient adherence, absent patients could be readily called back repeatedly and patients who had difficulty taking all their tablets at once could be called in stages; Frustrating, substantial and ongoing problems with video call quality were reported as well as the potential to not swallow the pills correctly

DOT: Directly Observed Treatment, MDR: multi-drug resistant; TB: Tuberculosis, VOT: Video observed treatment

For both patients and providers challenges were reported related to DOT provision. Lengthy travel time was mentioned as an obstacle for patients when DOT was provided in the health facility [54] and for providers when DOT was provided at the patient’s home or the community [39,44,57,65]. Also long duration of the home-DOT visits themselves and threats from the vicinity while visiting patients were reported to be obstacles for HCW [57]. Home-based DOT by a general nursing service implemented in Norway was perceived as humiliating and discriminating by some patients. They felt that there was little room to negotiate whether they consent to DOT. Some patients perceived inflexible DOT appointments as frustrating as it restricted their daily activities. Frequent changes in health care workers proving DOT hampered the establishment of a trustful relationship between the patients and providers [58].

#### Risk of bias within studies

Risk of bias was assessed for RCT (n = 2) [63,64] as well as for NRS with a control arm (n = 6) [30,35,39,48,65,66]. For both RCT there was an unclear risk of bias, i.e. insufficient information provided to determine the risk of bias, in more than one key domain (S3 Appendix). For all NRS a risk of bias was identified in one or more domains (S4 Appendix, S5 Appendix).

## Discussion

Our systematic review included 40 publications describing a variety of support interventions for TB patients in low-incidence countries; interventions that we categorized into DOT, SE support, PE support, HE support and other support. Only eight studies compared the intervention with a control group: six treatment supervision options (with reminders) and two HE support with reminders. In four of these studies the intervention(s) significantly increased treatment adherence and completion. Providing health education and reminders for medication intake and appointments [63,66], DOT by peers [64] and DOT at any location convenient to the patient [35] improved treatment adherence. DOT at the health care centre or the dispensary [48] and VOT [39,65] did not improve adherence. Qualitative and descriptive evaluations of patients’ experiences with treatment support showed that patients appreciate individualized support by nurses [56,60,62] or a “TB link worker” [43] including DOT, psycho-emotional as well as socio-economic support, and digital health interventions such as VOT as opposed to traditional forms of DOT [39,44,65] and blogging about TB [55]. These interventions may be beneficial in TB patient support in low-incidence countries.

Based on the evidence found in this review, combining health education and appointment/ medication reminders might be considered a best practice in TB patient support. The combined effect of health education and reminders has not been systematically reviewed yet. The importance of patient education in TB case management, however, was highlighted in another recent systematic review [69]; the success rate of treatment as well as the confirmed cure rate was significantly higher in the group receiving an intensive triad model (health education combined with support) compared to the control group. A review on reminder systems showed that appointment-reminders had a significant effect on treatment success and can be a valuable addition to other interventions [18]. A systematic review on digital technologies in TB treatment found that SMS reminders had no statistically significant effect on treatment adherence, while electronic medication monitors, i.e. medication boxes that record when the box is opened, increased the probability of cure in one observational study and reduced missed treatment doses in one trial [70].

Individualized support by nurses including PE support, SE support and possibly DOT might also be a beneficial intervention in low-incidence countries. An important finding from our Europe-wide survey among policy makers and nurse representatives also was that appointing a TB nurse to coordinate TB patient support (TB case management) may be considered a best practice in European low-incidence countries [71]. A systematic review on SE support and PE support of various forms found that most studies were conducted in in high-incidence settings, and that both SE and PE support improved treatment results [16]. SE support comprised direct enablers (food supplements, dietary advice, travel reimbursement, vouchers for local shops, clothing and hygiene kits), incentives (financial support, board games, newspapers and household goods) as well as legal support and assistance in procuring documentation for access to health care and social services. PE support included self-help groups, counselling, home visits, community groups and psychotherapy combined. Studies conducted in America before 2006 show that incentives and enablers, such as housing, monetary support, grocery coupons and transport reimbursement, improve treatment adherence [72–75].

Ideally, TB case management should be tailored to the individual patient and aligned with his or her expectations, i.e. patient-centred. Patient-centred care is much recommended in TB care in both low- and high-incidence countries [76]. A patient-centred approach includes exploring the patients’ needs for information, emotional needs, and life issues, finding common ground concerning the nature and the management of disease, enhancing prevention and health promotion as well as the relationship between the patient and the provider [77]. Patients receiving support as described in the publications included in this systematic review were frequently patients belonging to hard-to-reach groups such as immigrants, substance abusers, homeless and prisoners. A risk-group-focussed approach has been recommended by the WHO for countries progressing towards elimination [1]. The type and intensity of support needed is, however, not only dependent on the presence or absence of certain risk factors but also on individual patient characteristics [6]. Even within risk groups the need for support may differ between individuals and it is not one size fits all. In general, to allow a patient-centred approach, the patient’s needs are required to be assessed before implementation of treatment and support [77]. It may be essential to train health care workers in how to assess these needs and how to involve patients in the decision process.

Our review provided inconclusive evidence on the effectiveness of DOT in low-incidence countries but suggests that VOT might prioritized over traditional forms of DOT to improve patient and provider acceptance. A previous systematic review on DOT in both high and low-incidence settings concluded that TB cure and treatment completion did not improve substantially with DOT versus self-administered treatment [17]. Additionally, it showed that treatment completion or cure did not differ between patients who received DOT at home by family member or community health workers and patients who received DOT provided at the health facility by health workers [17]. In our review there was also no clear benefit of DOT for risk group patients. While peer DOT for substance abusers lead to positive effects on treatment adherence [64] while there was no correlation between DOT and treatment adherence in a population, in which DOT was given with priority to patients at risk of non-adherence [30]. A previous systematic review by Heuvelings et al. (2017) on TB treatment adherence interventions in hard-to reach populations in countries with low and medium TB incidence similarly concluded that DOT may improve adherence in only certain risk populations including homeless populations as well as migrants, prisoners and people living with HIV [20].

Similar to previously published literature [17,78–80], our review found that provision of traditional forms of DOT is surrounded with challenges for patients and providers, indicating a need for innovations. Only two studies included in this review found that patients appreciate DOT [56,58]; DOT combined with SE-, PE- and HE support for homeless patients reported patient-empowerment [56]. Migrants experienced DOT as an expression of care, especially when they were living very isolated [58]. Several other publications included in this review reported obstacles related to DOT such as patients feeling humiliated because DOT restricted their social life as they had to be at home during certain time slots for the DOT [58]. Also financial barriers through travel time and costs were reported, either for the patient when DOT is provided at the health facility or for the health care provider when DOT is provided at the patient’s home [39,44,54,57,65]. A substantial body of literature has already described obstacles and ethical concerns related to conventional DOT and questioned its’ effectiveness [17,78–80], and the need for new more flexible forms of DOT approaches has been expressed [80]. Few approaches addressing these obstacles related to more conventional DOT are identified by this review: only VOT and the use of peers. Use of peers increased adherence, which may be explained by reduced social distance between patient and provider [64]. VOT is described as an alternative to reduce travel time and costs for both patients and providers and to increase privacy [39,44,65]. As VOT can be provided at any place, if there is internet connection, it may also be easier to incorporate it into the patients’ everyday life [39,44,65]. The VOT studies conducted showed that adherence is similar to health facility- and home-based DOT [39,65]. The 2017 update of the WHO Guideline [76] for treatment of drug-susceptible TB and patient care states that VOT may replace DOT if the video communication technology is available and can be appropriately organized and operated by health care providers and patients. Further research will be needed to determine how VOT can best be combined with other treatment support resulting in satisfaction by both patients and health care workers, and in a positive treatment result.

Integrating digital health interventions in TB patients support may be beneficial for patients and providers, especially in low TB incidence countries. Both interventions identified in our review, VOT and blogging about MDR-TB, were appreciated by patients as well as providers. A recently published systematic review on the impact of digital health technologies on TB treatment showed that provision of SMS reminders and electronic medication monitors until now have been evaluated only in high-incidence countries [70] The implementation and evaluations of digital health in TB patient support interventions in low-incidence countries yet appear to be scarce. but. Especially for low-incidence, high-resource countries it may be useful to conduct further research on the value of digital health interventions in TB patient support. Digital health is expected to be increasingly implemented in TB patient support as information and communication technology is becoming more widely available and affordable, especially in high-income countries [11,81]. Digital health could be supportive to the need for more flexibility in patient support in general and in treatment supervision specifically. In addition to VOT, there are substantially more opportunities to use digital health solutions in patient support interventions such as a one-stop internet hub that links up to different services of relevance to TB care and health education [11]. These innovations may contribute to making support not only more flexible and more patient-friendly but possibly also more efficient and future-proof taking into account the decline of both patients and specialized TB health care providers in low incidence countries.

There were some limitations to this study. The literature review yielded only a small number of studies, especially studies including comparative quantitative outcomes of patient support interventions were scarce. Even fewer studies measure clinical outcomes such as cure and treatment failure which would be of greater importance to patient and providers compared to adherence measures only. Further, the studies were of low quality and studies and interventions described in this review were highly variable due to the design of the review aiming to provide an overview of patient support interventions implemented. Due to the small number of studies measuring quantitative outcomes and a large variation in interventions applied, outcomes measured and study populations among these studies could not be quantitatively synthesized and analysed.

Although the finding of this study are insufficient to provide recommendations on effective patients support in low incidence countries, our study identified some interventions that were effective and/ or appreciated by patients which would be useful to subject to further research. Furthermore, the framework we applied, with different patient support intervention categories (DOT, HE support, SE support, PE support and other), provides the opportunity to assess for the individual patients more systematically what categories of support are needed and to decide on the level of support e.g. on a scale of 1 (low), 2 (medium) and 3 (high). A standardised approach of defining the type and level of support to be provided will allow evaluating the complex nature of patient support. Further research is needed to determine for low-incidence countries how patients can best be involved in the decision-making process and to determine which support is most effective and efficient for these countries. This should be done taking into account the individual needs of the patients, risk group policy, the resources of the health care system and digital innovations.

### Conclusion

We provided an overview of support interventions currently implemented for TB patients in low-incidence countries and the evidence on its effects on treatment-related outcomes. Our review identified limited published evidence on effectiveness of patient support interventions in low-incidence countries, although we did identify a number of successful patient support interventions and possible best practices. Further research will be needed to verify these best practices and to determine which patient support is most effective and efficient.

## Supporting information

S1 Appendix Full text search term per data base. (DOCX)

S2 Appendix Overview of publications describing tuberculosis patient support in low-incidence countries. (DOCX)

S3 Appendix Risk of bias assessment of randomized control trials assessing the effect of patient support on treatment adherence–Cochrane collaborations tool for randomized controlled trials. (DOCX)

S4 Appendix Risk of bias assessment of case-control studies assessing the effect of patient support on treatment adherence–New-castle Ottawa scale for non-randomized studies. (DOCX)

S5 Appendix Risk of bias assessment of cohort studies, historically controlled studies and non-randomized controlled studies assessing the effect of patient support on treatment adherence–New-castle Ottawa scale for non-randomized studies. (DOCX)

S6 Appendix PRISMA checklist. (DOC)

S1 Fig PISMA flow diagram. (DOC)
