# Supplementary material for: Patient support for tuberculosis patients in low-incidence countries: A systematic review
Source: PLoS One. 2018 Oct 10;13(10):e0205433. doi: 10.1371/journal.pone.0205433 (PMC6179254; doi:10.1371/journal.pone.0205433)
Supplement: S1 Appendix — (DOCX) [file pone.0205433.s001.docx]

## S1 Appendix. Full text search term per data base

### PubMed

(tuberculosis [TIAB] OR tb[TIAB]) AND ((patient [TIAB] OR patients [TIAB] OR patient's [TIAB] OR nursing [TIAB] OR nurse[TIAB]) AND (support* [TIAB] OR assist*[TIAB] OR help[TIAB] OR intervention*[TIAB] OR program* [TIAB] OR “DOT” [TIAB] OR “directly observed treatment” [TIAB] OR “directly observed therapy” [TIAB] OR “SAT” [TIAB] OR “self-administered therapy”[TIAB])) AND (((medication[TIAB] OR therapy[TIAB] OR treatment [TIAB]) AND (adherence[TIAB] OR compliance [TIAB] OR completion [TIAB] OR "loss to follow-up"[TIAB] OR “lost to follow-up”[TIAB] OR default*[TIAB] OR conversion[TIAB] OR converted[TIAB])) OR qualit*[TIAB])

### Cumulative Index to Nursing and Allied Health and Literature and Social Science Citation Index

((tuberculosis OR tb) AND ((patient* OR nurs*) AND (support* OR assist* OR help OR intervention* OR program* OR “DOT” OR “directly observed treatment” OR “directly observed therapy” OR “SAT” OR “self-administered therapy")) AND (((medication OR therapy OR treatment) AND (adherence OR compliance OR completion OR "loss to follow-up" OR “lost to follow-up” OR default* OR conversion OR converted)) OR
