# Supplementary material for: Patient support for tuberculosis patients in low-incidence countries: A systematic review
Source: PLoS One. 2018 Oct 10;13(10):e0205433. doi: 10.1371/journal.pone.0205433 (PMC6179254; doi:10.1371/journal.pone.0205433)
Supplement: S2 Appendix — (DOCX) [file pone.0205433.s002.docx]

**S2 Appendix. Overview of publications describing tuberculosis patient support in low-incidence countries**

| **Study** | **Country** | **Study Type** | **Study Aim** | **Study Population** | **Support Categories Mentioned** | | | | |
| --- | --- | --- | --- | --- | --- | --- | --- | --- | --- |
|  |  |  |  |  | **TS** | **HES** | **SES** | **PES** | **Other** |
| **Studies quantitatively evaluating effects of patient support in comparison to a control group¤** | | | | | | | | | |
| Babalık et al., 2013 [1] | Turkey | Case-control study | Determine the factors influencing treatment outcomes and of the National Tuberculosis Program in relation to application of DOT | Adult TB patients with one year follow-up (n=905); 79% male; 45% belonging to the age group 18-35 years; 16% DR TB; 23% previously treated; 3% of non-Turkish origin; 24% co-morbidities; Cases: adverse treatment outcome (n=464), Controls: treatment outcome cured (n=441); 92% on DOT | DOT at health care centres (50%), dispensaries (20%) and other (30%), provided by health care workers (76%) and other (24%)* | - | - | - | - |
| Caylà et al., 2009 [2] | Spain | Prospective cohort study | Analyse anti-TB treatment adherence and fatality during standard TB treatments and identify factors associated with these event | Adult DS TB patients on standard anti-TB treatment (n=1424); 63% male; 40% aged 31-50 years; 88% PTB; 8% DR TB; 9% previously treated; 4% HIV-infected; 30% of foreign origin; 4% in confined institutions; 1.4% IDU; Exposed: patients on DOT^[[1]](#footnote-1)^ (n=140), Not exposed: patients on SAT (n=1284) | DOT^#¥^ | - | - | - | - |

**S2 Appendix. *(Continued)***

| **Study** | **Country** | **Study Type** | **Study Aim** | **Study Population** | **Support Categories Mentioned** | | | | |
| --- | --- | --- | --- | --- | --- | --- | --- | --- | --- |
|  |  |  |  |  | **TS** | **HES** | **SES** | **PES** | **Other** |
| **Studies quantitatively evaluating effects of patient support in comparison to a control group¤** | | | | | | | | | |
| Chaudhry et al., 2015 [3] | Saudi Arabia | Historical before-and-after study | Assess the effectiveness of the revised retrieval system (RRS) on non-compliance | Active PTB cases treated under DOT. Intervention phase 2005-2010 with RRS 2005-2010 (n=835); 65% male; 16% non-Saudi. Baseline phase 2002-2004 no RRS (n=501); 65% male; 66% non-Saudi | Out-patient DOT*^#¥^ | RRS: For all patients education at admission and discharge from the hospital; additionally, education at each OPD visit for substance-abusing patients^#^ | - | - | RRS: For all patients follow up after missed OPD appointments by national TB control nurse; additionally, reminders by national TB control nurses one day prior to appointments for substance-abusing patients |
| Chuck et al., 2016 [4] | USA | Non-randomized controlled trial | Determine completion rates of VOT in comparison with in-person DOT, feasibility, acceptability and resource and staffing needs | (DR) TB patients eligible for DOT IG: patients on VOT (n=61); 62% male; median age 36 years; 77% PTB; 43% DR TB; 10% HIV-positive; 92% not US-born. CG: patients on in-person DOT (n=329); 84% PTB; 8% DR; 60% male; median age 48 years; 5% HIV-infected; 89% not US-born | VOT: Live videos of the patients are via webcam-equipped computers^#^ | - | - | - | Missed VOT appointments followed up by phone calls and home visits^#^ |
| Clark et al., 2007 [5] | Turkey | Prospective randomized study | Assess the effect of a clinical pharmacist directed patient education program (EDU) on the therapy adherence compared to routine nursing care | First-time TB patients on first-line anti-TB drugs; IG: EDU (n=56); CG: no EDU (n=58) 100% male; mean age 38 years | - | EDU: Oral and written education by clinical pharmacist shortly before discharge from the hospital | - | - | EDU: Appointment reminders by clinical pharmacist |

**S2 Appendix. *(Continued)***

| **Study** | **Country** | **Study Type** | **Study Aim** | **Study Population** | **Support Categories Mentioned** | | | | |
| --- | --- | --- | --- | --- | --- | --- | --- | --- | --- |
|  |  |  |  |  | **TS** | **HES** | **SES** | **PES** | **Other** |
| **Studies quantitatively evaluating effects of patient support in comparison to a control group¤** | | | | | | | | | |
| King, Munsiff and Ahuja, 2010 [6] | USA | Retrospective cohort study | Review treatment outcomes of HIV- positive TB patients in New York City and determinants for treatment success | HIV-positive, first-time, Rifampicin-sensitive TB patients (n=2824); 70% male; 66% belong to the age group 19-45 years; 84% PTB; 29% non-US born; 13% homeless; Exposed: patient on DOT (n=1819), Not exposed: patients on SAT (n=592) | DOT at home, worksite or another location convenient to the patient ^#^ | - | - | - | - |
| Ricks et al., 2015 [7] | USA | Randomized intervention study | Compare treatment outcomes using two different types of DOT outreach workers | Substance abusing active TB patient for which DOT was prescribed; IG (n=48), CG (n=46); 61% male; mean age 41 years; 10% previous TB; 13% HIV-infected; 16% incarcerated in the past 6 months; 35% in unstable housing | Enhanced DOT: DOT provided by peers in a two-person mixed-sex team ^¥^ | - | - | - | - |
| Wade et al., 2012 [8] | Australia | Retrospective cohort study with CEA | Compare the effectiveness (as measured by the proportion of appointments missed) of in-person DOT versus VOT; determine the cost-effectiveness, acceptability, usability and sustainability of VOT | TB patients who had received VOT/DOT; IG (n=58): 55% male; 36 % aged 20-29 years; 98% foreign-born. CG (n=70); 60% male; 23% aged 20-29 years; 84% foreign-born | VOT: DOT via desktop videophones and a call centre operating 24/7 and set up by a community nursing service | - | - | - | - |

**S2 Appendix. *(Continued)***

| **Study** | **Country** | **Study Type** | **Study Aim** | **Study Population** | **Support Categories Mentioned** | | | | |
| --- | --- | --- | --- | --- | --- | --- | --- | --- | --- |
|  |  |  |  |  | **TS** | **HES** | **SES** | **PES** | **Other** |
| **Studies quantitatively evaluating effects of patient support without allowing for comparison of effects to a control group¤** | | | | | | | | | |
| Charokopos et al., 2013 [9] | Greece | Case-control study | Determine the effect of "modified DOT” (MDOT) on TB treatment outcomes, number of contacts tested for LTBI and number of contacts started on treatment in comparison to a SAT | Cases: newly diagnosed TB patients (n=13) and close contacts (n=30); 77% male; mean age 45 years; 23% belonging to (ethnic) minority group. Controls: past-treated TB patients (n=41) and close contacts (n= 111); 68% male; mean age 67 years; 0% belonging to (ethnic) minority groups^[[2]](#footnote-2)^ | MDOT: Treatment supervision by GP during nine home visits, every 20 days | MDOT: Health education by the GP for the patient and household members during the visits | - | - | - |
| Craig et al., 2008 [10] | UK | Case series | Develop a social outreach model of care including a link worker (TBLW) for marginalized groups with TB | Adult TB/LTBI patients referred on the basis of social need to TBLW (n=100); 62% male; median age 32 years; 63% of non-European origin | DOT at the drug dependency unit (DDU), at the pharmacy or the TB clinic* | - | TBLW: helps patients with challenging health and social care needs to access community services | - | - |
| Escudero et al., 2006 [11] | Spain | Case series | Evaluate the results of the treatment of non-HIV-infected MDR-TB patients | HIV-negative MDR PTB patients (n=25); 92% male; mean age 42 years; 88% previously treated; 100% received DOT+PES | In-patient DOT by nurses | - | - | Psychological support and counselling by repeated clinical interviews on need and difficulties related to treatment adherence during hospitalisation and during out-patient follow-up | - |

**S2 Appendix. *(Continued)***

| **Study** | **Country** | **Study Type** | **Study Aim** | **Study Population** | **Support Categories Mentioned** | | | | |
| --- | --- | --- | --- | --- | --- | --- | --- | --- | --- |
|  |  |  |  |  | **TS** | **HES** | **SES** | **PES** | **Other** |
| **Studies quantitatively evaluating effects of patient support without allowing for comparison of effects to a control group¤** | | | | | | | | | |
| Ferrer et al., 2010 [12] | USA | Case series | Report treatment outcomes among MDR-TB patients born in Mexico and treated along the US-Mexican border under a binational TB control project (Programa Juntos) | MDR-TB patients (n=48); 73% male, mean age 45 years; 77% history of TB; 33% concomitant medical conditions; 0% HIV-infected; 100% born in Mexico; 100% on DOT | Out-patient DOT by social workers^¥^ | - | - | - | - |
| Garfein et al., 2015 [13] | USA | Case series | Determine feasibility, acceptability, and potential efficacy of VOT in a high- and low-income setting | Adult newly diagnosed DS TB patients treated under VOT (n=43 in San Diego; n=9 in Tijuana); 50% male; mean age 37 years | VOT: Patients upload videos of themselves taking the medication to a cloud via a smart phone app^#^ | - | - | - | Daily text message reminders (one before doses due and one after the expected video had not been received) |
| Jit et al., 2011 [14] | UK | Retrospective cohort study with CEA | Evaluate the cost-effectiveness of the Find and Treat Service for diagnosing and managing hard to reach individuals with active TB | Hard to reach individuals (e.g. homeless, substance abusing, imprisoned) a with active PTB screened or managed by the Find and Treat service (n=48), passively presenting controls (n=252) ^[[3]](#footnote-3)^ | - | Awareness raising events by Find and Treat Service supported by peer workers | - | Company to appointments by Find and Treat Service staff; home visits to reduce the risk of loss to follow-up | - |

**S2 Appendix. *(Continued)***

| **Study** | **Country** | **Study Type** | **Study Aim** | **Study Population** | **Support Categories Mentioned** | | | | |
| --- | --- | --- | --- | --- | --- | --- | --- | --- | --- |
|  |  |  |  |  | **TS** | **HES** | **SES** | **PES** | **Other** |
| **Studies quantitatively evaluating effects of patient support without allowing for comparison of effects to a control group¤** | | | | | | | | | |
| Luzzati et al., 2011 [15] | Italy | Case series | Evaluate a prolonged hospitalisation programme to improve early outcome of TB treatment in high risk patients | Adult patients admitted to referral TB Centre for high risk (DR TB, foreign born, illegal immigrant, previously treated, IDU, HIV infected or in a social and/or familiar condition not assuring good adherence to treatment) with positive smear culture-confirmed PTB (n=122); 62.3% males; 100% on DOT | In-patient DOT, subsequently out-patient DOT ^#¥^ | - | - | - | - |
| Mejuto et al., 2010 [16] | Spain | Retrospective cohort study | Realize a retrospective study of the characterization, results and effectiveness of DOTS in the regional health area of Santiago de Compostela | TB patients who received DOTS treatment (n=253); 77% male; mean age 40 years; 88% PTB; 31% previously treated; 3% MDR-TB; 33% social dystocia and psychiatric illness; 29% alcohol abusers; 17% drug abusers; 12% HIV-infected; 2% immigrants | DOT at TB unit, health centre, social services, family, DDU, school, hospital^#^ | - | - | - | - |

**S2 Appendix. *(Continued)***

| **Study** | **Country** | **Study Type** | **Study Aim** | **Study Population** | **Support Categories Mentioned** | | | | |
| --- | --- | --- | --- | --- | --- | --- | --- | --- | --- |
|  |  |  |  |  | **TS** | **HES** | **SES** | **PES** | **Other** |
| **Studies quantitatively evaluating effects of patient support without allowing for comparison of effects to a control group¤** | | | | | | | | | |
| Pursnani et al., 2014 [17] | USA | Case-control study nested in a retrospective cohort study | Compare patients undergoing court-ordered detention for TB treatment and time-matched control TB patients on outpatient DOT | Patients undergoing court-ordered detention for TB treatment (n=79) ^[[4]](#footnote-4)^; 65% male; mean age 42 years; 100% PTB; 19% DR TB; 61% HIV-infected; 38% non-US born; 52% drug abusers; 38% alcohol abusers; 42% homeless; 25% mental illness; 18% history of incarceration.Patients on outpatient DOT (n=70); 77% male; mean age 44 years; 89% PTB; 9% DR TB; 15% HIV-infected; 93% non-US born; 13% drug abusers; 11% alcohol abusers; 7% homeless; 9% mental illness; 6% history of incarceration | Out-patient DOT*^#¥^ | - | - | - | - |
| **Studies qualitatively assessing different aspects of patient support** | | | | | | | | | |
| Bender et al., 2011 [18] | Canada | Interpretive phenomenology | Understand the nature of TB nurses’ relational work | Female nurses (n=9) and their clients (n=24); 58% male | DOT by nurses at patients’ homes, nurses’ cars, the street and other public settings* | Nurses repeatedly explain and clarify treatment plan | Incentives (such as grocery vouchers and public transit tokens)* | Nurses build rapport, encourage adherence without being authoritarian | - |
| Craig and Zumla, 2015 [19] | UK | Interview study | Describe the social context of adherence to treatment in marginalized groups | Patients from a major TB centre (n=17); 71% male; mean age 44 years; 41% non-UK born; 53% on DOT | DOT at the DDU, the pharmacy in conjunction with methadone and at the hostel via outreach workers* | - | - | Outreach workers accompany patients to appointments* | Outreach workers provide appointment reminders* |

**S2 Appendix. *(Continued)***

| **Study** | **Country** | **Study Type** | **Study Aim** | **Study Population** | **Support Categories Mentioned** | | | | |
| --- | --- | --- | --- | --- | --- | --- | --- | --- | --- |
|  |  |  |  |  | **TS** | **HES** | **SES** | **PES** | **Other** |
| **Studies qualitatively assessing different aspects of patient support** | | | | | | | | | |
| Gerrish, Naisby and Ismail, 2013 [20] | UK | Focused ethnography | Explore experiences of the diagnosis and management of TB from the perspective of Somali patients living in the UK and healthcare professionals involved in their care | Healthcare practitioners with experience of caring for Somali TB patients (n=18). Somalis who had received TB treatment in the UK (n=14); 64% male; 71% PTB; 100% received support from TB specialist nurses | - | - | Somali health care workers and TB nurses help patients to access other health and welfare services | - | - |
| Horter et al., 2014 [21] | Multi-national | Interview study | Identify potential risks and benefits associated with blogging to determine whether social media had a role to play in supporting patients with MDR-TB | MDR patient bloggers (n=5); MSF project staff closely involved with the bloggers (n=8); Stakeholders: WHO European Region TB specialists (n=2) and members of staff from MSF headquarters (n=5) | - | - | - | Blogging about MDR TB treatment | - |
| Kawatsu et al., 2013 [22] | Japan | Interview study | Explore the changes experienced by homeless TB patients and discuss the possible role of PHC-based DOTS treatment in effecting these changes | Ex-homeless TB patients who completed DOTS-based treatment at Shinjuku City PHC (n=18); 100% male; median age 59 years; 56% co-morbidities | DOT by nurses at the public health centre | - | Provision of food and drinks when patients come for DOT; nurses consult social welfare offices and other organizations | Nurses build rapport, address concerns, congratulation ceremony for successfully completed treatment | - |

**S2 Appendix. *(Continued)***

| **Study** | **Country** | **Study Type** | **Study Aim** | **Study Population** | **Support Categories Mentioned** | | | | |
| --- | --- | --- | --- | --- | --- | --- | --- | --- | --- |
|  |  |  |  |  | **TS** | **HES** | **SES** | **PES** | **Other** |
| **Studies qualitatively assessing different aspects of patient support** | | | | | | | | | |
| Mtui and Spence, 2014 [23] | UK | Interview study | Explore the views and experiences of National Health Service (NHS) board TB nurses and consultants in public health medicine in relation to models of TB service delivery employed in their respective NHS boards in Scotland | TB specialist nurses (n=6); health protection specialist nurse (n=2); respiratory specialist nurse (n=5); consultants in public health medicine (n=5) | DOT at GP practices, in pharmacies for substance-abusers, on the streets / public bars by TB nurses for homeless patients | Nurses talk about TB and provide leaflets* | Nurses assist in accessing social care while delivering DOT; provide incentives for some cases, bring people to the clinic* | Nurses build rapport with patients, support in coping with the treatment, perform home visits* | - |
| Sagbakken, Bjune and Frich, 2011 [24] | Norway | Interview study | Explore patients’ and health professionals’ views and experiences with DOT | Health professionals (n=20), TB patients on DOT (n=22); 45% male; 45% in age group 19-25 years; 100% of non-Norwegian origin | DOT by homebased nursing services | - | - | - | - |
| Searle, Park and Littleton, 2007 [25] | New Zealand | Community-based ethnography | Document and analyse the nature of the process of TB care in older European (Pakeha) TB patients | European TB patients in the Auckland region (n=8); 63% male; median age 60 years; 75% PTB; 40% previous TB; 63% on DOT | DOT at home by public health nurses* | - | Nurses eased structural constrains by arranging housing, food and transport | Nurses provided moral support and encouragements | - |
| Shimamura et al., 2010 [26] | Japan | Interview study | Describe the support provided by Japanese public health nurses (PHN) to high-risk TB patients | PHNs (n=11); 100% female, patient cases described by the PHN (n=11); 82% male; age range 30-80 years; 100% Japanese; 36% homeless; 18% older persons with dementia | DOT by public health nurses*^¥^ | Nurses explained TB and co-morbidities to the patient and contacts, for patients with limited intelligence using a comic book or picture-story | Nurses ensure physical place for homeless patients to receive medications, link patients with welfare service, build a support system for the future, including housing, food, or job training | Nurses build rapport, encourage patients | Pill case provided for one patient with dementia who hoped to take her medicine independently |

**S2 Appendix. *(Continued)***

| **Study** | **Country** | **Study Type** | **Study Aim** | **Study Population** | **Support Categories Mentioned** | | | | |
| --- | --- | --- | --- | --- | --- | --- | --- | --- | --- |
|  |  |  |  |  | **TS** | **HES** | **SES** | **PES** | **Other** |
| **Studies mentioning patient support provision** | | | | | | | | | |
| Anger et al., 2010 [27] | USA | Retrospective cohort study | Examine treatment outcomes, incidence, management and predictors of adverse events among a cohort of MDR TB patients treated with linezolid as part of salvage regimens | MDR TB patients who received treatment with linezolid for ≥1 month (n=16); 38% male; median age 38 years; 94% PTB; 75% co-morbidity; 100% hospitalized or on DOT for the first 6 months; 88% hospitalized or on DOT for the duration of therapy | Out-patient DOT^*#¥^ | - | - | - | - |
| Banerjee et al., 2008 [28] | USA | Case series | Determine the extent of XDR TB within California and describe characteristics and outcomes of XDR TB | XDR TB patients (n=18); 100% PTB; median age 42 years; 50% prior TB; 83% of non-US origin; 6% homeless; 6% incarcerated; 0% AIDS; 0% IDU; 59% on DOT | DOT*^#¥^ | - | - | - | - |
| Ehman, Flood and Barry, 2014 [29] | USA | Retrospective cohort study | Determine whether there were differences in care provided by public health departments and private medical providers | Adult, culture-positive TB patients without extra-pulmonary disease (n=4606); 63% male; 35% belonging to age group 45-64 years; 14% DR TB; 6% previous TB; 3% HIV-infected, 83% non-US born; 15% homeless, alcohol or drug use; 7% not on DOT | DOT*^#¥^ | - | - | - | - |

**S2 Appendix. *(Continued)***

| **Study** | **Country** | **Study Type** | **Study Aim** | **Study Population** | **Support Categories Mentioned** | | | | |
| --- | --- | --- | --- | --- | --- | --- | --- | --- | --- |
|  |  |  |  |  | **TS** | **HES** | **SES** | **PES** | **Other** |
| **Studies mentioning patient support provision** | | | | | | | | | |
| García-García et al., 2011 [30] | Spain | Prospective cohort study | To identify the differential TB characteristics within the immigrant population with respect to natives in Spain | Adult TB patients treated with standard regimen (n=1490); Natives (n=1063); 65% male; 38% belonging to age group 31-50 years; 86% PTB; 4% DR TB; 5% HIV-infected; 3% homeless or incarcerated; 9% previously treated; 2% IDU; 9% on DOT. Immigrants (n=427); 60% male; 48% belonging to age group 18-30 years; 79% PTB; 7% DR TB; 4% HIV-infected; 5% homeless or incarcerated; 9% previously treated; 1% IDU; 14% on DOT | DOT*^#¥^ | - | - | - | - |
| Guglielmi et al., 2006 [31] | Switzerland | Cohort study | Determine the operational treatment outcome in the canton of St Galen, where administrative treatment monitoring is functional | Newly diagnosed TB patients (n=112); 66% male; mean age 42 years; 79% PTB; 1% previously treated; 3% HIV-infected; 63% of non-Swiss origin; 6% on DOT | DOT at the doctor’s office, the local pharmacy* or shelters for asylum seekers^#^ | - | - | - | - |

**S2 Appendix. *(Continued)***

| **Study** | **Country** | **Study Type** | **Study Aim** | **Study Population** | **Support Categories Mentioned** | | | | |
| --- | --- | --- | --- | --- | --- | --- | --- | --- | --- |
|  |  |  |  |  | **TS** | **HES** | **SES** | **PES** | **Other** |
| **Studies mentioning patient support provision** | | | | | | | | | |
| Katsuda et al., 2015 [32] | Japan | Cross-sectional | Describing the role of public health centres (PHC) in TB control | TB patients in Japan (n=8424 in 2010) | For homeless people, alcohol or drug dependents, recurrent cases and patients with interruption history: every day mandatory visit of patient to a clinic, hospital, pharmacy or public health centre for DOT by medical staff of clinic or hospital, pharmacist of pharmacy, or public health nurse at PHC | - | Incentives such as city sponsored apartments and public assistance | - |  |
| Khan et al., 2011 [33] | Canada | Retrospective cohort study | Assess trends in TB among all homeless persons in Toronto who had a diagnosis of active TB during 1998–2007 | Homeless, active TB patients from Toronto (n=102); 89% male; median age 47 years; 32% not Canada-born; 77% PTB; 12% HIV-infected; 11% psychiatric disease; 32% chronic alcohol abuse; 13% IDU; DOT, SES and adherence support other than DOT for most patients | Out-patient DOT *^#¥^ | - | Small incentives and enablers (e.g. food vouchers or cash) | Company by public health staff to all clinic visits | - |
| Krueger et al., 2010 [34] | USA | Retrospective cohort study with CEA | Examine the cost-effectiveness of videophone use as an alternative to in-person DOT | TB patients who received VOT from 2002 through 2006 (n=57) | VOT^#^ | - | - | - | - |

**S2 Appendix. *(Continued)***

| **Study** | **Country** | **Study Type** | **Study Aim** | **Study Population** | **Support Categories Mentioned** | | | | |
| --- | --- | --- | --- | --- | --- | --- | --- | --- | --- |
|  |  |  |  |  | **TS** | **HES** | **SES** | **PES** | **Other** |
| **Studies mentioning patient support provision** | | | | | | | | | |
| Kurt et al., 2012 [35] | Turkey | Retrospective cohort study | Determine TB trends, to evaluate effectiveness of regional TB control studies and to detect problems in the control studies carried out from 2004−2008 in Mersin | TB patients in study region (n=1776): in 2008 n=313; 23.3* male; 34.7* aged 55–64 years; 8.6* previously treated; 97% on DOTS  *per 100,000 population | DOT*^#¥^ | - | - | - | - |
| Pevzner et al., 2010 [36] | USA | Case series | Investigate a cluster of TB cases among persons using methamphetamines in Snohomish County, Washington | Outbreak cases (n=10); 70% male; median age 35 years; 80% PTB; 0% HIV-infected; 100% US-born; 80% methamphetamine users; 100% on DOT | DOT at a needle exchange program, a homeless shelter and on the streets by staff from the substance abuse and sexually transmitted disease unit | - | Incentives (coupons, restaurant gift cards, cash, incentives tailored to special circumstances), enablers (gas cards, taxi service, hotel room for one homeless patient) | - | - |
| Pritchett et al., 2009 [37] | USA | Case series | Review the application of legal procedures and to evaluate their effectiveness in the management of non-adherent TB patients | TB patients non-adherent to treatment and regular clinic visits for examination and monitoring for toxicity (n=39); 44% male; 35% HIV-infected; 64% substance-abusers; 23% homeless; 100% on DOT | DOT at clinic, home, school or workplace^#^ | Education on TB and importance of DOT and medical follow-up during initial patient assessment interview by TB control case managers | Incentives e.g. bus tokens, enablers, counselling by a full-time social worker, social services referrals and placement functions | - | - |

**S2 Appendix. *(Continued)***

| **Study** | **Country** | **Study Type** | **Study Aim** | **Study Population** | **Support Categories Mentioned** | | | | |
| --- | --- | --- | --- | --- | --- | --- | --- | --- | --- |
|  |  |  |  |  | **TS** | **HES** | **SES** | **PES** | **Other** |
| **Studies mentioning patient support provision** | | | | | | | | | |
| Rubado et al., 2008 [38] | USA | Costing study | Establish the average cost per patient of nurse case management for TB | TB patients in Multnomah and Marion Counties (n=42); 56% male; median patient age group 40–44 years; 70% PTB; 7% HIV-infected; 61% non-US nationality; 92% on DOT | DOT by nurse case manager (NCM) or outreach worker^*¥^ | - | NCM identifies and attempts to overcome social and financial obstacles to treatment adherence, provides financial incentives, organizes treatment of co-morbidities | NCM identifies and attempts to overcome psychological obstacles to treatment adherence | - |
| Story et al., 2007 [39] | UK | Cross-sectional study with 12 months follow-up | Determine the point prevalence of TB disease in different groups and to examine risk factors for smear positivity, drug resistance, treatment adherence, loss to follow-up and use of DOT | TB patients living in London who were or should have been on treatment on 1 July 2003 (n=1941); 55% male; 47% aged 30-59 years; 10% previous TB; 80% foreign born; 13% problem drug users; 4% imprisoned; 6% homeless; 25 % ever on DOT during treatment | DOT^*#¥^ | - | - | - | - |
| Van Altena et al., 2015 [40] | Netherlands | Retrospective cohort study | Assess the treatment outcomes of MDR-TB patients over a period of 10 years | MDR-TB patients (n=113); 61% male; median age 29 years; 31% previously treated; 12% HIV-infected; 96% of non-Dutch origin; 100% on in-patient DOT and unknown percentage on out-patient DOT | In-patient DOT by nursing staff at the TB centre; Out-patient DOT by nurse from municipal health service*^¥^ | - | - | - | - |

^*^target patients not specified ^#^provider not specified ^¥^DOT location not specified

CG: control group, CEA: cost effectiveness analysis, DDU: drug dependency unit, DOT: directly observed treatment, DR: drug resistant, DS: drug susceptible, GP: general practitioner, HES: health educational support, HIV: Human Immunodeficiency Virus, IDU: injecting drug user, IG: intervention group, LTBI: latent TB infection, MDR TB: multi-drug resistant TB, MSF: Médecins Sans Frontières, NHS: National Health Service, OPD: out-patient department, PES: psycho-emotional support, PHC: public health centre, PHN: public health nurse, PTB: pulmonary TB, RRS: revised retrieval system, SAT: self-administered treatment, SES: socio-economic support, TB: Tuberculosis, TBLW: TB link worker, TS: Treatment Supervision, VOT: video-observed treatment

**References**

[1] Babalık A, Kılıçaslan Z, Kızıltaş S, Gencer S, Ongen G. A retrospective case-control study, factors affecting treatment outcomes for pulmonary tuberculosis in istanbul, Turkey. Balkan Med J 2013;30:204–10. doi:10.5152/balkanmedj.2013.005.

[2] Caylà JA, Rodrigo T, Ruiz-Manzano J, Caminero JA, Vidal R, García JM, et al. Tuberculosis treatment adherence and fatality in Spain. Respir Res 2009;10:121. doi:10.1186/1465-9921-10-121.

[3] Chaudhry LA, Al-Tawfiq J, Ba-Essa E, Robert AA. Low rate of non-compliance to antituberculous therapy under the banner of directly observed treatment short course (DOTS) strategy and well organized retrieval system: A call for implementation of this strategy at all DOTS centers in Saudi Arabia. Pan Afr Med J 2015;21:1–5. doi:10.11604/pamj.2015.21.267.6280.

[4] Chuck C, Robinson E, Macaraig M, Alexander M, Burzynski J. Enhancing management of tuberculosis treatment with video directly observed therapy in New York City. Int J Tuberc Lung Dis 2016;20:588–93. doi:10.5588/ijtld.15.0738.

[5] Clark PM, Karagoz T, Apikoglu-Rabus S, Izzettin FV. Effect of pharmacist-led patient education on adherence to tuberculosis treatment. Am J Heal Pharm 2007;64:497–506. doi:10.2146/ajhp050543.

[6] King L, Munsiff SS, Ahuja SD. Achieving international targets for tuberculosis treatment success among HIV-positive patients in New York City. Int J Tuberc Lung Dis 2010;14:1613–20.

[7] Ricks PM, Hershow RC, Rahimian A, Huo D, Johnson W, Prachand N, et al. A randomized trial comparing standard outcomes in two treatment models for substance users with tuberculosis 2015;19:326–32.

[8] Wade VA, Karnon J, Eliott JA, Hiller JE. Home Videophones Improve Direct Observation in Tuberculosis Treatment: A Mixed Methods Evaluation. PLoS One 2012;7:1–13. doi:10.1371/journal.pone.0050155.

[9] Charokopos N, Tsiros G, Foka A, Voila P, Chrysanthopoulos K, Spiliopoulou I, et al. Modified directly observed treatment for tuberculosis versus self-administered therapy: An observational study in rural Greece. Rural Remote Health 2013;13:1–10.

[10] Craig GM, Booth H, Hall J, Story A, Hayward A, Goodburn A, et al. Establishing a new service role in tuberculosis care: The tuberculosis link worker. J Adv Nurs 2008;61:413–24. doi:10.1111/j.1365-2648.2007.04498.

[11] Escudero E, Peña JM, Vázquez JJ, Ortega A. Multidrug-resistant tuberculosis without HIV infection : Int J 2006;10:409–14.

[12] Ferrer G, Acuna-Villaorduna C, Escobedo M, Vlasich E, Rivera M. Outcomes of multidrug-resistant tuberculosis among binational cases in El Paso, Texas. Am J Trop Med Hyg 2010;83:1056–8. doi:10.4269/ajtmh.2010.10-0145.

[13] Garfein RS, Collins K, Muñoz F, Moser K, Cerecer-Callu P, Raab F, et al. Feasibility of tuberculosis treatment monitoring by video directly observed therapy: A binational pilot study. Int J Tuberc Lung Dis 2015;19:1057–64. doi:10.5588/ijtld.14.0923.

[14] Jit M, Stagg HR, Aldridge RW, White PJ, Abubakar I. Dedicated outreach service for hard to reach patients with tuberculosis in London: observational study and economic evaluation. Bmj 2011;343:d5376–d5376. doi:10.1136/bmj.d5376.

[15] Luzzati R, Confalonieri M, Cazzadori A, Della Loggia P, Cifaldi R, Fabris C, et al. Prolonged hospitalisation for immigrants and high risk patients with positive smear pulmonary tuberculosis. Monaldi Arch Chest Dis - Pulm Ser 2011;75:141–5. doi:10.4081/monaldi.2011.229.

[16] Mejuto B, Tunez V, del Molino MLP, Garcia R. Characterization and evaluation of the directly observed treatment for tuberculosis in Santiago de Compostela (1996-2006). Risk Manag Healthc Policy 2010;3:21–6.

[17] Pursnani S, Srivastava S, Ali S, Leibert E, Rogers L. Risk factors for and outcomes of detention of patients with TB in New York City: An update: 2002-2009. Chest 2014;145:95–100. doi:10.1378/chest.13-0324.

[18] Bender A, Peter E, Wynn F, Andrews G, Pringle D. Welcome intrusions: An interpretive phenomenological study of TB nurses’ relational work. Int J Nurs Stud 2011;48:1409–19. doi:10.1016/j.ijnurstu.2011.04.012.

[19] Craig GM, Zumla A. The social context of tuberculosis treatment in urban risk groups in the United Kingdom: A qualitative interview study. Int J Infect Dis 2015;32:105–10. doi:10.1016/j.ijid.2015.01.007.

[20] Gerrish K, Naisby A, Ismail M. Experiences of the diagnosis and management of tuberculosis: A focused ethnography of somali patients and healthcare professionals in the UK. J Adv Nurs 2013;69:2285–94. doi:10.1111/jan.12112.

[21] Horter S, Stringer B, Venis S, Du Cros P. “I can also serve as an inspiration”: A qualitative study of the TB&Me blogging experience and its role in MDRTB treatment. PLoS One 2014;9. doi:10.1371/journal.pone.0108591.

[22] Kawatsu L, Sato N, Ngamvithayapong-Yanai J, Ishikawa N. Leaving the street and reconstructing lives: Impact of DOTS in empowering homeless people in Tokyo, Japan. Int J Tuberc Lung Dis 2013;17:940–6. doi:10.5588/ijtld.12.0503.

[23] Mtui L, Spence W. An exploration of NHS staff views on tuberculosis service delivery in Scottish NHS boards. J Infect Prev 2014;15:24–30.

[24] Sagbakken M, Bjune GA, Frich JC. Humiliation or care? A qualitative study of patients’ and health professionals’ experiences with tuberculosis treatment in Norway. Scand J Caring Sci 2012;26:313–23. doi:10.1111/j.1471-6712.2011.00935.x.

[25] Searle A, Park J, Littleton J. Alliance and compliance in tuberculosis treatment of older Pakeha people in Auckland, New Zealand. Int J Tuberc Lung Dis 2007;11:72–7.

[26] Shimamura T, Taguchi A, Kobayashi S, Nagata S, Magilvy JK, Murashima S. The strategies of japanese public health nurses in medication support for high-risk tuberculosis patients. Public Health Nurs 2013;30:370–8. doi:10.1111/phn.12010.

[27] Anger HA, Dworkin F, Sharma S, Munsiff SS, Nilsen DM, Ahuja SD. Linezolid use for treatment of multidrug-resistant and extensively drug-resistant tuberculosis, New York City, 2000-06. J Antimicrob Chemother 2010;65:775–83. doi:10.1093/jac/dkq017.

[28] Banerjee R, Allen J, Westenhouse J, Oh P, Elms W, Desmond E, et al. Extensively Drug‐Resistant Tuberculosis in California, 1993–2006. Clin Infect Dis 2008;47:450–7. doi:10.1086/590009.

[29] Ehman M, Flood J, Barry PM. Tuberculosis treatment managed by providers outside the public health department: Lessons for the affordable care act. PLoS One 2014;9. doi:10.1371/journal.pone.0110645.

[30] García-García JM, Blanquer R, Rodrigo T, Caylà JA, Caminero JA, Vidal R, et al. Social, clinical and microbiological differential characteristics of tuberculosis among immigrants in Spain. PLoS One 2011;6. doi:10.1371/journal.pone.0016272.

[31] Guglielmi S, Barben J, Horn L, Schoch OD. Administrative monitoring of tuberculosis treatment in Switzerland. Int J Tuberc Lung Dis 2006;10:1236–40.

[32] Katsuda N, Hirosawa T, Reyer J a, Hamajima N. Roles of Public Health Centers () in Tuberculosis Control in Japan. Nagoya J Med Sci 2015;77:19–28.

[33] Khan K, Rea E, Mcdermaid C, Stuart R, Chambers C, Wang J, et al. Active tuberculosis among homeless persons, Toronto, Ontario, Canada, 1998-2007. Emerg Infect Dis 2011;17:357–65. doi:10.3201/eid1703.100833.

[34] Krueger K, Ruby D, Cooley P, Montoya B, Exarchos A, Djojonegoro BM, et al. Videophone utilization as an alternative to directly observed therapy for tuberculosis [Short communication]. Int J Tuberc Lung Dis 2010;14:779–81.

[35] Kurt AO, Sasmaz T, Bugdayci R, Oner S, Yapici G, Ozdemir O. A five year retrospective surveillance; monitoring and evaluation for the regional tuberculosis control programme in Mersin, Turkey, 2004-2008. Cent Eur J Public Health 2012;20:144–9.

[36] Pevzner ES, Robison S, Donovan J, Allis D, Spitters C, Friedman R. Tuberculosis Transmission and Use of Methamphetamines in Snohomish County , WA , 1991 – 2006. Am J Public Health 2010;100:2481–6. doi:10.2105/AJPH.2009.162388.

[37] Pritchett EN, Schlossberg D, Beck J, Dickman B. Legal intervention for non-adherent patients in the treatment of tuberculosis. Int J Tuberc Lung Dis 2009;13:323–7.

[38] Rubado DJ, Choi D, Becker T, Winthrop K, Schafer S. Determining the cost of tuberculosis case management in a low-incidence state. Int J Tuberc Lung Dis 2008;12:301–7.

[39] Story A, Murad S, Roberts W, Verheyen M, Hayward AC. Tuberculosis in London: the importance of homelessness, problem drug use and prison. Thorax 2007;62:667–71. doi:10.1136/thx.2006.065409.

[40] Altena R Van, Vries G De, Haar CH, Lange WCM De, Hof S Van Den, van Altena R, et al. Highly successful treatment outcome of multidrug-resistant tuberculosis in the Netherlands , 2000 – 2009. Int J Tuberc Lung Dis 2015;19:406–12. doi:10.5588/ijtld.14.0838.

1. Patients with high risk of low adherence (intravenous drug users, homeless, prisoners) [↑](#footnote-ref-1)
2. Publication does not provide treatment outcomes for controls [↑](#footnote-ref-2)
3. Treatment outcome data based on modelling [↑](#footnote-ref-3)
4. Patients undergoing court-ordered detention for TB treatment are not considered a comparison group for this systematic review as court-ordered detention in not considered patient support [↑](#footnote-ref-4)
