# Supplementary material for: Patient support for tuberculosis patients in low-incidence countries: A systematic review
Source: PLoS One. 2018 Oct 10;13(10):e0205433. doi: 10.1371/journal.pone.0205433 (PMC6179254; doi:10.1371/journal.pone.0205433)
Supplement: S3 Appendix — (DOCX) [file pone.0205433.s003.docx]

# S3 Appendix. Risk of bias assessment of randomized control trials assessing the effect of patient support on treatment adherence – Cochrane collaborations tool for randomized controlled trials

| **Study** | **Random sequence generation** | **Allocation concealment** | **Blinding of participants** | **Blinding of personnel** | **Blinding of outcome assessment** | **Incomplete data (per outcome)** | **Selective reporting** | **Other sources of bias** |
| --- | --- | --- | --- | --- | --- | --- | --- | --- |
| Clark et al., 2007 [1] | No information provided | No information provided | No information provided | Personnel was aware of their allocation status after randomization | No information on blinding of outcome assessors | 1. Attended visit percentage: Loss to follow-up is an outcome 2. Positive isoniazid test result percentage: 9% lost to follow-up in intervention group, 10% in control group; loss to follow-up ≤10% and not stat. significantly different between the two groups (p>0.05) 3. Mean ± S.D. consumed medication percentage: No information provided | No information on selective reporting | NA |
| *Review authors’ judgement* | *No judgment possible, unclear risk of selection bias.* | *No judgment possible, unclear risk of bias* | *No judgment possible, unclear risk of performance bias.* | *Low risk of performance bias as the outcome is not likely to be influenced due to lack of personnel blinding* | *No judgment possible, unclear risk of detection bias.* | 1. *Attended visit percentage: low risk of attrition bias* 2. *Positive isoniazid test result percentage: Low risk of attrition bias* 3. *Mean ± S.D. consumed medication percentage: No judgment possible, unclear risk of attrition bias* | *No judgment possible, unclear risk of reporting bias* | *NA* |
| Ricks et al., 2015 [2] | Computer generated random assignment | Sealed, opaque, consecutively numbered and ordered envelopes | Participants were aware of their allocation status after randomization | Personnel was aware of their allocation status after randomization | No information on blinding of outcome assessors | 1. Treatment completion: No information provided 2. Treatment adherence [mean nr of interruptions]: No data for 6% of the intervention group and 10% of the control group; loss to follow-up ≤10% and not stat. significantly different between the two groups (p>0.05) 3. Treatment adherence [mean length of interruptions] No data for 6% of the intervention group and 10% of the control group; loss to follow-up ≤10% and not stat. significantly different between the two groups (p>0.05) | No information on selective reporting | NA |
| *Review authors’ judgement* | *Low risk of selection bias* | *Low risk of bias* | *Low risk of performance bias as the outcome is not likely to be influenced due to lack of patient blinding* | *Low risk of performance bias as the outcome is not likely to be influenced due to lack of personnel blinding* | *No judgment possible, unclear risk of detection bias* | 1. *No judgment possible, unclear risk of attrition bias* 2. *Low risk of attrition bias* 3. *Low risk of attrition bias* | *No judgment possible, unclear risk of reporting bias* | *NA* |

**References**

[1] Clark PM, Karagoz T, Apikoglu-Rabus S, Izzettin FV. Effect of pharmacist-led patient education on adherence to tuberculosis treatment. Am J Heal Pharm 2007;64:497–506. doi:10.2146/ajhp050543.

[2] Ricks PM, Hershow RC, Rahimian A, Huo D, Johnson W, Prachand N, et al. A randomized trial comparing standard outcomes in two treatment models for substance users with tuberculosis 2015;19:326–32.
