# Supplementary material for: Patient support for tuberculosis patients in low-incidence countries: A systematic review
Source: PLoS One. 2018 Oct 10;13(10):e0205433. doi: 10.1371/journal.pone.0205433 (PMC6179254; doi:10.1371/journal.pone.0205433)
Supplement: S4 Appendix — (DOCX) [file pone.0205433.s004.docx]

**S4 Appendix. Risk of bias assessment of case-control studies assessing the effect of patient support on treatment adherence** **– New-castle Ottawa scale for case-control studies**

| **Study** | **Adequate Case Definition** | **Representativeness of the Cases** | **Selection of Controls** | **Definition of Controls** | **Comparability of Cases and Controls on the Basis of the Design or Analysis** | **Ascertainment of Exposure** | **Same method of ascertainment for cases and controls** | **Non-Response Rate** |
| --- | --- | --- | --- | --- | --- | --- | --- | --- |
| Babalık et al., 2013 [1] | Internationally accepted WHO definitions were used | Random selection of cases from National Tuberculosis database | Random selection of controls from same National Tuberculosis database | Cases have new occurrence of adverse treatment outcome | Odds ratios adjusted for age, sex and previous treatment | Data was collected from medical records in the Tuberculosis National Database | Yes | Not mentioned |
| *Review authors’ judgement: 6 stars* | *Standardized case definitions available in database, one star awarded* | *Consecutive representative series of cases, one star awarded* | *Unknown whether the controls are derived from the same population as the cases, no stars awarded* | *Control with previous occurrences of adverse treatment outcome are not excluded, one star awarded* | *Odds ratio for the exposure adjusted to confounders, cases and controls considered comparable on each variable used in the adjustment, two stars awarded* | *No secure record, no stars awarded* | *One star awarded* | *No stars awarded* |

**References**

[1] Babalık A, Kılıçaslan Z, Kızıltaş S, Gencer S, Ongen G. A retrospective case-control study, factors affecting treatment outcomes for pulmonary tuberculosis in istanbul, Turkey. Balkan Med J 2013;30:204–10. doi:10.5152/balkanmedj.2013.005.
