# Supplementary material for: Patient support for tuberculosis patients in low-incidence countries: A systematic review
Source: PLoS One. 2018 Oct 10;13(10):e0205433. doi: 10.1371/journal.pone.0205433 (PMC6179254; doi:10.1371/journal.pone.0205433)
Supplement: S5 Appendix — (DOCX) [file pone.0205433.s005.docx]

**S5 Appendix. Risk of bias assessment of cohort studies, historically controlled studies and non-randomized controlled studies assessing the effect of patient support on treatment adherence** **– New-castle Ottawa scale for non-randomized studies**

| **Study** | **Representative of the exposed cohort** | **Selection of non-exposed cohort** | **Ascertainment of exposure** | **Demonstration that outcome of interest was not present at the start of the study** | **Comparability of cohorts on the basis of the design or analysis** | **Assessment of outcome** | **Was follow-up long enough for outcomes to occur** | **Adequacy of follow-up of cohorts** |
| --- | --- | --- | --- | --- | --- | --- | --- | --- |
| Caylà et al. 2009 [1] | No description of the sub-cohort receiving DOT | No description of the sub-cohort not receiving DOT | Electronic diary made available through a computerised application | Patients were followed up prospectively from initiation of treatment; when outcome of interest (i.e. treatment outcome) could not have been present | No descriptions of sub-cohorts | Electronic diary made available through a computerised application | Treatment outcome for all subjects available | Treatment outcome for all subjects available |
| *Review authors’ judgement: 5 stars* | *No description of the derivation of the cohort, no star awarded* | *No description of the derivation of the non-exposed cohort, no star awarded* | *Secure record, one star awarded* | *Demonstration that outcome of interest was not present at start of study, one star awarded* | *No adjustment for differences between the groups, no star awarded.* | *Record linkage, one star awarded* | *Follow-up long enough for outcomes to occur, one star awarded* | *Complete follow up - all subjects accounted for, one star awarded* |
| Chaudhry et al, 2015 [2] | Patients of open pulmonary TB admitted and treated under DOTS at the TB centre of Dammam Medical Complex | Historical controls from the same location. | Case files and treatment cards | Intervention was provided throughout the treatment | NA | Case files and treatment cards | Treatment outcome for all subjects available | Treatment outcome for all subjects available |
| *Review authors’ judgement: 5 stars* | *Selected group of users, no star awarded* | *Drawn from a different source, no star awarded* | *Secure record, one star awarded* | *Demonstration that outcome of interest was not present at start of study, one star awarded* | *No adjustment for differences between the groups, no star awarded.* | *Record linkage, one star awarded* | *Follow-up long enough for outcomes to occur, one star awarded* | *Complete follow up - all subjects accounted for, one star awarded* |
| Chuck et al., 2016 [3] | Patients were not eligible if they had a history of non-adherence | Compared to patients receiving in-person DOT, VOT patients were younger, more likely to have resistance to at least one anti-TB medication, and more likely to be treated at one of the NYC Department of Health and Mental Hygiene TB clinics | Records of DOT workers | Patients were enrolled prospectively and were followed for 9 months or until discharge, e.g., completed treatment, lost to care, or refused to continue treatment. | NA | Records of DOT workers | Patients were followed for 9 months or until discharge, e.g., completed treatment, lost to care, or refused to continue treatment | Total patients not eligible to complete treatment 62/329 DOT (19%), 12/61 VOT (20%) |
| *Review authors’ judgement: 4 stars* | *Selected group of users, no star awarded* | *Drawn from a different source, no star awarded* | *Secure record, one star awarded* | *Demonstration that outcome of interest was not present at start of study, one star awarded* | *No adjustment for differences between the groups, no star awarded.* | *Record linkage, one star awarded* | *Follow-up long enough for outcomes to occur, one star awarded* | *Follow up rate < 10% and no description of those lost, no star awarded* |

**S5 Appendix. *(continued)***

| **Study** | **Representative of the exposed cohort** | **Selection of non-exposed cohort** | **Ascertainment of exposure** | **Demonstration that outcome of interest was not present at the start of the study** | **Comparability of cohorts on the basis of the design or analysis** | **Assessment of outcome** | **Was follow-up long enough for outcomes to occur** | **Adequacy of follow-up of cohorts** |
| --- | --- | --- | --- | --- | --- | --- | --- | --- |
| King, Munsiff and Ahuja [4] | No description of the sub-cohort receiving DOT | No description of the sub-cohort not receiving DOT | Registered NYC TB patients | Outcome of interest (i.e. treatment success/ death during treatment) could not have been present before exposure of interest (i.e. DOT) | NA | Registered NYC TB patients | Treatment outcome for all subjects available | Treatment outcome for all subjects available |
| *Review authors’ judgement: 5 stars* | *No description of the derivation of the cohort, no star awarded* | *No description of the derivation of the non-exposed cohort, no star awarded* | *Secure record, one star awarded* | *Demonstration that outcome of interest was not present at start of study, one star awarded* | *No adjustment for differences between the groups, no star awarded.* | *Record linkage, one star awarded* | *Follow-up long enough for outcomes to occur, one star awarded* | *Complete follow up - all subjects accounted for, one star awarded* |
| Wade et al., 2012 [5] | Patients under VOT in South Australia likely represent average VOT patient | Patients in South Australia during the same period | Records of health care workers | Intervention was provided throughout the treatment | NA | Records of health care workers | Treatment outcome for all subjects available | Treatment outcome for all subjects available |
| *Authors’ judgement: 7 stars* | *Somewhat representative of the average VOT patient in the community, one star awarded* | *Drawn from the same community as the exposed cohort, one star awarded* | *Secure record, one star awarded* | *Demonstration that outcome of interest was not present at start of study, one star awarded* | *No adjustment for differences between the groups, no star awarded.* | *Record linkage, one star awarded* | *Follow-up long enough for outcomes to occur, one star awarded* | *Complete follow up - all subjects accounted for, one star awarded* |

**References**

[1] Caylà JA, Rodrigo T, Ruiz-Manzano J, Caminero JA, Vidal R, García JM, et al. Tuberculosis treatment adherence and fatality in Spain. Respir Res 2009;10:121. doi:10.1186/1465-9921-10-121.

[2] Chaudhry LA, Al-Tawfiq J, Ba-Essa E, Robert AA. Low rate of non-compliance to antituberculous therapy under the banner of directly observed treatment short course (DOTS) strategy and well organized retrieval system: A call for implementation of this strategy at all DOTS centers in Saudi Arabia. Pan Afr Med J 2015;21:1–5. doi:10.11604/pamj.2015.21.267.6280.

[3] Chuck C, Robinson E, Macaraig M, Alexander M, Burzynski J. Enhancing management of tuberculosis treatment with video directly observed therapy in New York City. Int J Tuberc Lung Dis 2016;20:588–93. doi:10.5588/ijtld.15.0738.

[4] King L, Munsiff SS, Ahuja SD. Achieving international targets for tuberculosis treatment success among HIV-positive patients in New York City. Int J Tuberc Lung Dis 2010;14:1613–20.

[5] Wade VA, Karnon J, Eliott JA, Hiller JE. Home Videophones Improve Direct Observation in Tuberculosis Treatment: A Mixed Methods Evaluation. PLoS One 2012;7:1–13. doi:10.1371/journal.pone.0050155.
