# Supplementary material for: Patient support for tuberculosis patients in low-incidence countries: A systematic review
Source: PLoS One. 2018 Oct 10;13(10):e0205433. doi: 10.1371/journal.pone.0205433 (PMC6179254; doi:10.1371/journal.pone.0205433)
Supplement: S1 Fig — (DOC) [file pone.0205433.s007.doc]

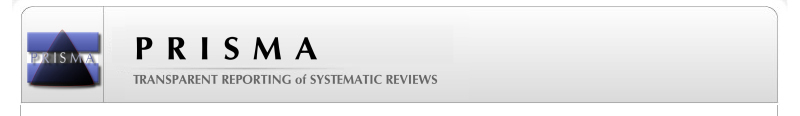
**PRISMA 2009 Flow Diagram**

**Screening**

**Included**

**Eligibility**

**Identification**

Records identified through database searching
(n = 2411 )

Additional records identified through other sources
(n = 23)

Records after duplicates removed
(n = 1875)

Records screened
(n = 1875 )

Records excluded
(n = 1778)

Full-text articles assessed for eligibility
(n = 97 )

Full-text articles excluded
(n = 57)

- No tuberculosis patient support described (n = 44)
- No low-incidence country (n = 12)
- Publication in Japanese (n = 1)

Studies included in qualitative synthesis
(n = 97)

Studies included in quantitative synthesis (meta-analysis)
(n = 40)
